# Suppression of Mitochondrial Complex I Influences Cell Metastatic Properties

**DOI:** 10.1371/journal.pone.0061677

**Published:** 2013-04-22

**Authors:** Xuelian He, Aifen Zhou, Hao Lu, Yong Chen, Guochang Huang, Xin Yue, Peiwei Zhao, Yanxiang Wu

**Affiliations:** 1 Central Laboratory, Wuhan Medical and Healthcare Center for Women and Children, Hubei, China; 2 Department of Healthcare, Wuhan Medical and Healthcare Center for Women and Children, Hubei, China; 3 Institute of Molecular and Cell Biology, A*STAR (Agency for Science, Technology and Research), Biopolis, Singapore; Stony Brook University, United States of America

## Abstract

Despite the fact that mitochondrial dysfunction has an important role in tumorigenesis and metastasis, the underlying mechanism remains to be elucidated. Mitochondrial Complex I (NADH:ubiquinone oxidoreductase) is the first and the largest protein complex of the mitochondrial electron-transport chain (ETC),which has an essential role in maintaining mitochondrial function and integrity. In this study, we separately knocked down two subunits of mitochondrial complex I, GRIM-19 or NDUFS3, and investigated their effects on metastatic behaviors and explored the possible mechanisms. Our data showed that stable down-modulation of GRIM-19 or NDUFS3 decreased complex I activity and reactive oxygen species (ROS) production; led to enhanced cell adhesion, migration, invasion, and spheroid formation; and influenced the expressions of extracellular matrix (ECM) molecules and its related proteins. We also observed that the expressions of GRIM-19, NDUFS3, and ECM elements were correlated with invasive capabilities of breast cancer cell lines. These results suggest that inhibition of complex I affects metastatic properties of cancer cells, and mitochondrial ROS might play a crucial role in these processes by regulating ECM.

## Introduction

Metastasis or the spread of cancer is the primary cause of death in most patients with malignancy and understanding the underlying molecular mechanisms represents one of the great challenges in exploratory cancer research. Metastasis is a multi-stage process involving cancer cell motility, invasion, intravasation, transit in the blood or lymph, extravasation and proliferation at a new site [1]. When cancer cells become metastatic, invade and migrate into surrounding tissues, they change their behaviors in interaction with extracellular matrix (ECM) and surrounding cells [2]. Tumor cell adhesion to ECM proteins is mediated by integrins and the binding of integrins to ECM proteins activates signaling pathways that regulate gene expression, cell growth, cell adhesion, spreading, migration and invasion [3]–[4].

Mitochondria are subcellular organelles, whose well-known function is to produce adenosine triphosphate (ATP) through the oxidative phosphorylation system (OXPHOS). Five multi-subunit complexes (I-V) and two additional mobile electron carriers-coenzyme Q10 and cytochome *c* are responsible for oxidative phosphorylation. In addition, mitochondria also perform essential function in the regulation of cell death, cell signaling, innate immunity and autophagy through key signaling mediators such as reactive oxygen species (ROS). Given the crucial role of mitochondria in these cellular pathways, defects in mitochondria function contribute to a number of human disorders, including cancer development and metastasis.

Complex I is the largest and most complicated enzyme that catalyzes the first step in electron transfer chain and is also one of the main sites of ROS production [5]. However, whether complex I subunits are associated with cancer metastasis and their contributions to the pathogenesis of cancer have not been fully defined. In this study, we separately inhibit mitochondrial complex I activity by suppressing its two subunits, GRIM-19 and NDUFS3, using siRNA technique and determine the role of complex I in cancer metastasis.

## Results

### Knockdown of GRIM-19 and NDUFS3 Reduces Mitochondrial Respiratory Chain (RC) Complex I Activity

**Figure 1:**
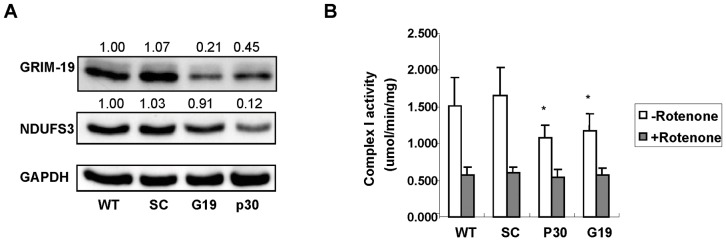
Knockdown of GRIM-19 and NDUFS3 Suppresses Mitochondrial Complex I Activity. The knockdown efficiency of *GRIM-19* or *NDUFS3* was assessed by densitometric analysis of GRIM-19 and NDUFS3 bands on western blot using GAPDH as loading control (A). The complex I activity was tested by measuring absorbance at a wavelength of 340 nm using spectrophotometer with NADH as the substrate. The rotenone-sensitive NADH oxidation rate which represents the complex I activity was calculated by subtracting the NADH oxidation rate in the presence of rotenone from the total NADH oxidation rate in the absence of rotenone (B). Asterisks indicate a p-value of ≤0.05 (*) as determined by Student's T-test.

In order to determine if mitochondrial complex I has a role in metastasis-related cancer behavior, two subunits of complex I, GRIM-19 or NDUFS3, were separately knocked down using siRNA in Hela cells. After establishing stable cells, the knockdown efficiency was examined by western blot analysis. The relative protein expressions of GRIM-19 and NDUFS3 in wildtype (WT), siRNA-*GRIM-19* cells (G19), siRNA-*NDUFS3* cells (p30), and a control transfected with scrambled sequence for *NDUFS3* gene (SC) were calculated by densitometric analysis by using β-actin as loading control. The GRIM-19 expression was inhibited by ∼80% and NDUFS3 protein expression was suppressed by ∼90%, compared to WT and SC (Figure 1A). It has been noticed that knockdown of *NDUFS3* also led to a loss of GRIM-19 expression, and knockdown of *GRIM-19* reduced NDUFS3 level, as observed previously [6], which suggested a mutual effect of these two subunit proteins. The mitochondrial complex I activity in these cells was determined by measuring NADH oxidation rate by spectrophotometer *in vitro*. As shown in Figure 1B, the overall NADH oxidation rate in G19 and p30 cells was significantly decreased in comparison with that in the WT and SC cells (p<0.05). To determine the contribution of the complex I activity in the overall NADH oxidation reaction, the cells were treated with rotenone, a complex I inhibitor. In the presence of rotenone, the NADH oxidation rate was decreased to similar levels in all four cell lines. The rotenone-sensitive NADH oxidation rate which represents the complex I activity was inhibited about 43% and 49% in G19 and p30 cells, respectively, compared with SC cells (Figure 1B).

### Suppression of GRIM-19 or NDUFS3 Induced Epithelial–mesenchymal Transition (EMT) Phenotype and Increased Cell Adhesion, Migration, Invasion and Spheroid Formation

**Figure 2:**
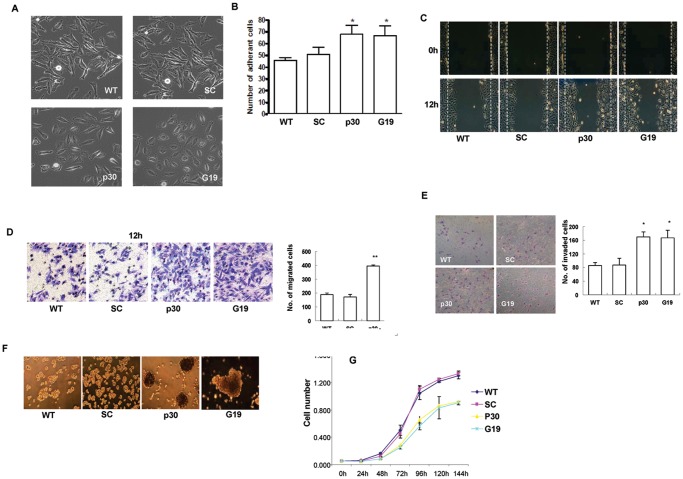
Knockdown of GRIM-19 and NDUFS3 in Cells Induced EMT-Like Phenotypes and Inhibited Cell Proliferation. Cells were seeded onto plates coated with FN and cultured for indicated times at 37°C for one day in CO_2_ incubator. After washing using PBS and fixing using 3.7% paraformaldehyde, the cell morphology was examined under a phase-contrast microscope (A). Serum-starved Hela cells (5×10^5^) were seeded onto collagen-coated plates and cultured for 30 min. After washing with serum free media, the attached cells were counted and compared (B). Migration assay was assessed using wound healing and transwell methods, respectively. The healing of wound was imaged at 12 hr in wound-healing assay (C) and the migrated through the filter membrane of transwell were stained with 0.4% crystal violet and imaged (D). Cell invasion was tested by using Matrigel-coated transwell filters and the cells invaded through Matrigel and filter were imaged (E). Spheroid formation was evaluated by seeding 5000 cells/ml of each cell lines into 1% agarose gel-coated plates. The cells were cultured at normal culture conditions and the spheroid formation was monitored at appropriate time points (F). Knockdown GRIM-19 and NDUFS3 reduce cell proliferation in normal culture conditions (G). Asterisks indicate a p-value of ≤0.05 (*) or ≤0.01(**) as determined by Student's T-test.

After silencing *GRIM-19* or *NDUFS3* gene, we noticed the cells lost epithelial morphology and acquired mesenchymal characteristics, such as cell scattering, lost colonial morphology and increased lamellipodia (Figure 2A). We also investigated whether there are any functional consequences on cancer progression and metastasis potential after inhibiting complex I activity. Firstly, we performed a cell-matrix adhesion assay. The results showed that both *GRIM-19* or *NDUFS3* knockdown cells exhibited significantly higher cell-matrix adhesion capability in comparison with WT and SC cells (p<0.01)(Figure 2B). In addition, we performed wound healing and transwell migration assays to evaluate the cell motility. Our results showed the cell migration rates were significantly enhanced in G19 and p30 cells in wound closure (Figure 2C) and transwell migration assay (Figure 2D) compared to WT and SC cells. Interestingly, G19 and p30 cells had a more than 2-fold increase in invasion compared to the control cells as measured with Matrigel-transwell assay (Figure 2E) (p<0.05).

In addition to the cell-matrix interaction, we also examined cell-cell adhesion by spheroid formation assay in three-dimensional culture. We observed that round and tight spheroids were formed at 48 hr in G19 and p30 cells, while no obvious spheroid was observed in WT and SC at the same time point (Figure 2F). In order to exclude the possibility that the increased migration and invasion in knockdown cells were due to increased cell proliferation, we examined the cell proliferation rate. In contrast to the increased rate of migration and invasion, the proliferation rates in the G19 or p30 cells were significantly reduced (p<0.01) (Figure 2G). These observations demonstrated that the suppression of GRIM-19 or NDUFS3 in Hela cells promoted cell adhesion, migration, invasion and spheroid formation, and inhibited the cell proliferation.

### Suppression of GRIM-19 and NDUFS3 Enhanced Levels of FN, Integrins, and N-cadherin

**Figure 3:**
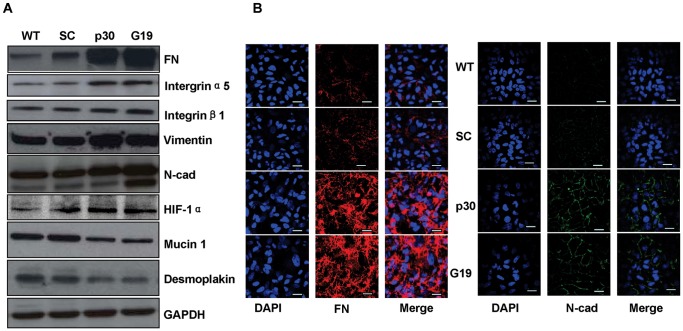
Molecular Profiling of EMT-Associated Proteins. Western blot analysis of FN, its receptor integrins α5 and β1, N-cadherin, Vimentin, Mucin 1, Desmoplakin and HIF1a in WT, SC, G19 and p30 Hela cells (A). FN (B, left panel) and N-cadherin (B, right panel) expression in monolayer adhesion culture environment was assessed using immunofluorescence. Scale bar = 20 µm.

To investigate the possible mechanism involved in the changes in cell adhesive and invasive behavior in the *GRIM-19* or *NDUFS3* knockdown cells, we isolated RNA from SC, G19, and p30 cells and conducted gene expression analysis using Affymetrix GeneChip Human Genome U133 Plus 2.0 GeneChip arrays. After analyzing our microarray data using GeneGo software (http://www.genego.com), 10 most significantly altered canonical pathways were identified. Of the 10 pathways, 6 pathways are involved in extracellular matrix (ECM) remodeling, intergin outside-in signaling, integrin-mediated cell adhesion, and migration (detailed data not shown). To validate microarray data, selected genes and proteins were examined using semi-quantitative RT-PCR and/or western blot and/or (fibronectin(FN), integrin α5 and β1, vimentin, N-cadherin (N-cad) (E-cadherin is undetectable in Hela cells (data not shown)), HIF-1α, Mucin 1, and Desmoplakin (Figure 3A). FN and N-cad were further verified by immunofluorescence staining (Figure 3B). Among these proteins, FN and its receptors, integrin α5 and β1, were increased. It is well-known that FN is also an EMT marker. Another two EMT markers, N-cadherin and vimentin, were also increased in G19 and p30 cells compared to WT and SC cells, while epithelial markers, mucin 1 and desmoplakin, were decreased. Consistent with the cellular morphological changes, such as cell scattering, loss of cell-cell contacts, these EMT markers indicated that silencing *GRIM-19* or *NDUFS3* induced EMT in Hela cells. In addition to these ECM and related components and EMT markers, HIF1α, an important microenviromental factor linking mitochondrial dysfunction with cancer tumorigenesis and metastasis, was also increased under normoxic conditions. Its downstream gene products, VEGF and TGF-β are also increased. Collectively, these results suggested the inhibition of mitochondrial complex I could could activate transcription factors, such as HIF1α, then trigger EMT, a centrally important mechanism for the progression of cancer to a metastatic stage, with the help of intracellular signaling networks, such as integrin outside-in signaling.

### Knockdown of GRIM-19 or NDUFS3 Promoted ***R***OS Generation which Correlated with the Expression Levels of Adhesion Proteins and EMT Phenotype

**Figure 4:**
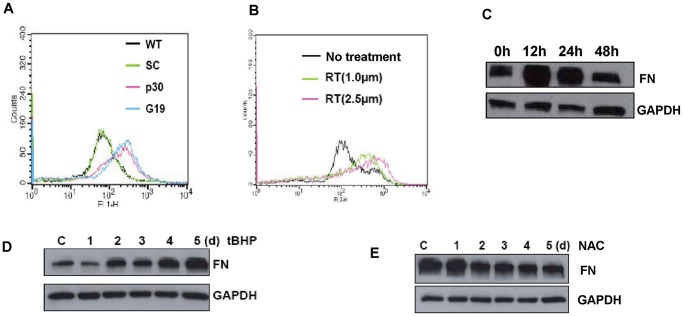
Partial Inhibition of Complex I by Knockdown *GRIM-19*, *NDUFS3* or by Chemical Inhibitor Increased ROS Generation thereby Increasing the FN and N-cadherin Levels. Knockdown of GRIM-19 or NDUFS3 increases ROS generation as compared to the SC or WT cells. ROS was measured by staining cells with H_2_DCFH-DA followed by FACS scan (A). Rotenone (RT) treatment induces the ROS production in the WT cells in a dose dependent manner (B). The effect of ROS on FN expression was assessed by Western blot after treating SC cells with rotenone (2.5 µM) in (C) or 200 µM tBHP in (D) at indicated days. The ROS scavenger NAC can decrease the FN expression level of G19 Hela cells (E).

It has been demonstrated that deficiency of mitochondrial RC leads to increased production of ROS, and the increase is primarily a consequence of the reduction of the complex I activity [7]–[8]. Furthermore, ROS serve as signaling molecules and influence many basic cellular functions, such as proliferation, apoptosis, migration, and adhesion [9]–[12]. Thus, we postulated that the generation of ROS could be increased by inhibiting complex I activity in the *GRIM-19* or *NDUFS3* knockdown cells and the increased ROS may be the major cause of the observed alterations in cell behavior. In order to verify this hypothesis, the ROS level was measured by flow cytometry using fluorescent dye H_2_DCFH-DA. As shown in Figure 4A, G19 and p30 knockdown cells produced higher ROS compared with the control WT and SC cells. As a control, treatment of WT cells with an complex I inhibitor, rotenone, resulted in a dose-dependent increase of the ROS-generating cells (Figure 4B), and a concomitant increase of the FN expression (Figure 4C).

To further verify the link between the ROS production and the expression of adhesion proteins, we treated the SC cells with 200 µM tert-Butylhydroperoxide (tBHP), a ROS generation agent, and found FN expression level elevated from day 2 to day 5 (Figure 4D). In contrast, treatment of G19 cells with 5 mM N-acetylcysteine (NAC), an antioxidant, resulted in an obvious decrease in the FN level from day 2 to day 5 (Figure 4E). More interestingly, the NAC treatment could suppress the increased cell motility in the G19 knockdown cells, but no such effect in the WT cells (data not shown). These data demonstrated a major role of ROS in up-regulation of the adhesion proteins and the EMT phenotypes in the knockdown cells.

### Differential Expression of GRIM-19, NDUFS3, HIF-1α and Adhesion Proteins in Metastatically Different Breast Cancer Cell Lines

**Figure 5:**
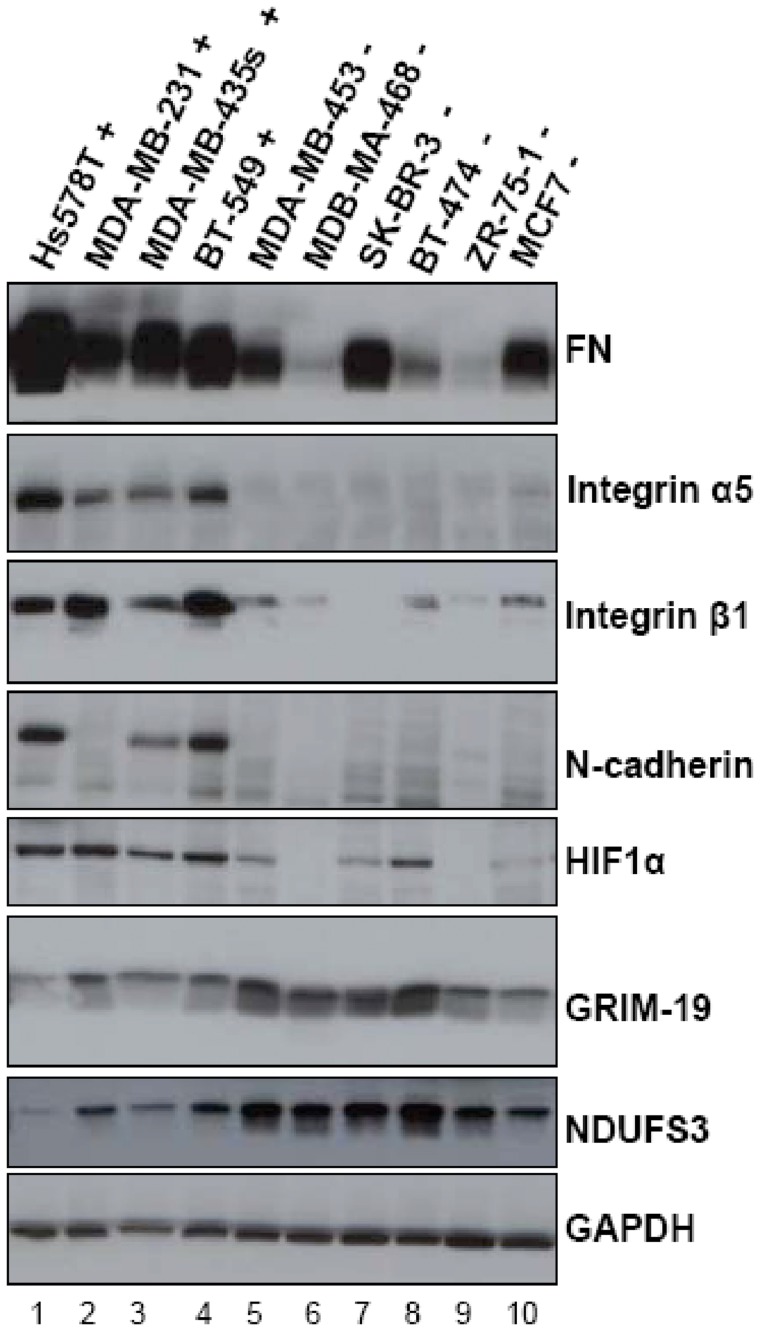
Evidence for the Loss of GRIM-19 or NDUFS3 in Relation to Metastatic Potential from Different Mammary Cell Lines. Elevated protein levels of FN, Integrins, N-cadherin and HIF1α were observed in highly metastatic breast cancer cell lines while decreased protein level of GRIM-19 and NDUFS3 is observed in these cell lines. The lysates from 10 different breast cancer cell lines were subjected to Western blot using indicated primary antibodies and GAPDH as a loading control.

As indicated by the results described above, after suppressing *GRIM-19* or *NDUFS3*, the expressions of HIF-1α, vimentin, FN, integrin α5 and β1, and N-cadherin expression were increased, and cells exhibited increased invasive properties. Thus, we examined whether these protein expression levels were correlated with invasive properties in highly invasive (Hs578T, MDA-MB-231, MDA-MB-435s and BT-549) and weakly invasive (ZR-75-1, MDA-MB-453, MDB-MA-468, SK-BR-3, BT-474) breast cancer cell lines. As shown in Figure 5, the general expression profile exhibited higher levels of HIF-1α, FN, intergrin α5, intergrin β1, and N-cadherin in most cell lines with highly invasive feature (lanes 1–4) compared with those with low invasion capacity (lanes 5–10). Conversely, levels of GRIM-19 and NDUFS3 were reduced in these highly invasive cell lines compared with these weakly invasive cell lines. As a control, the level of GAPDH was consistent in all cell lines. These results indicate that GRIM-19 and NDUFS3 expression levels are negatively, whereas HIF-1α, FN, integrin α5 and β1, and N-cadherin are positively, correlated with the invasive property in these breast cancer cell lines, which is in agreement with the observations in the G19 and p30 cells. Our results suggest a correlation between the mitochondrial complex I dysfunction and the invasive properties of malignant tumor cells.

## Discussion

In this study, we aimed to investigate whether the impairment of mitochondrial complex I affects invasive phenotypes of malignant tumor cells and if it does, what the underlying mechanisms are. Our data lead to two major findings. (i) Complex I activity can affect cancer metastatic potentials by mediating cell-ECM and cell-cell interaction, and promoting EMT. (ii) Complex I deficiency-induced ROS generation may play a critical role in these processes by regulating ECM and its related protein expression.

The Complex I consists of at least 46 subunits encoded by both mitochondrial and genomic DNA giving it a unique dual genetic control. In this study, we knocked down either of its two nuclear encoded subunits (GRIM-19 or NDUFS3) by siRNA approach. GRIM-19 is the product of a cell death regulatory gene induced by interferon-β (IFN-β) and retinoic acid in human cancer cell lines [13]. Our laboratory identified that GRIM-19 is not only a subunit of complex I but also a negative regulator of STAT3 [14]. Thus, GRIM-19 may be a potential anti-oncogenic protein. *NDUFS3*, the other gene we knocked down, encodes a 30-kDa subunit of mitochondrialcomplex I and is predicted to carry out essential aspects of complex I function. Furthermore, NUDFS3 was recently reported to be decreased in mammary carcinoma [15]. Thus, the protein levels of these subunits, perhaps also the other nuclear-encoded subunits, in malignant tumors can serve as biomarkers for tumor growth and metastatic transition.

After suppressing these two subunits, as expected, the complex I activity was decreased and the knockdown one of these two subunits also suppressed protein level of the other, which provide an efficient way to study the complex I functions. Our results showed that knockdown of *GRIM-19* or *NDUFS3* induced EMT, enhanced cell adhesion, migration, invasion, and spheroid formation, but inhibited cell proliferation. Consistent with these phenotypes observed, the expression levels of genes and proteins involved in ECM remodeling, integrin-mediated cell adhesion, integrin outside-in signaling pathways were significantly changed, such as FN, integrin α5, N-cadherin, and vimentin after analyzed by cDNA microarray assay and further validated by western blot. Among these proteins, the change in FN expression was most dramatic. Importantly, we found that the expression levels of GRIM-19 and NDUFS3 were decreased level in the highly invasive breast cancer cell lines in comparison with that in the weakly invasive lines by screening of a panel of breast cancer cells (Figure 5), which support the role of mitochondrial complex I and even mitochondrial respiratory chain (RC) in the tumor cell invasion. In addition, decreased expression levels of GRIM-19 and NDUFS3 were found in renal cell carcinomas samples by western blot and RT-PCR analysis [16]–[17]. NDUFS3 was also reported to be decreased in breast cancer carcinoma [18]. Thus, the expression levels of these mitochondrial complex I subunits, perhaps also other mitochondrial DNA encoded subunits, in the tumors can serve as biomarkers for tumor growth and metastatic transition.

The role of mitochondria in tumorigenesis has been linked to their ROS production. Under normal physiological conditions, about 1 to 3% of total mitochondrial oxygen consumed is incompletely reduced and leads to ROS production. As mitochondrial RC is a permanent source of ROS, cell has an effective antioxidant systems to nullify the toxic effects of the ROS and other free radicals [19]. Excessive ROS production can cause mtDNA mutations, which may lead to mitochondrial RC dysfunction that could contribute to the onset of many diseases including neoplasia. When mitochondria function is affected, mitochondrial ROS production can be increased and hence trigger oncogenic pathways. As expected, we observed an enhanced ROS level in the *GRIM-19* or *NDUFS3* knockdown cells. ROS as a signal can affect expressions and functions of many genes. In relation to the EMT phenotype, we detected significant increase of several cell adhesion proteins and related proteins, such as FN, intergrins, N-cadherin, vimentin and HIF1α in the knockdown cells (Figure 3A).

As FN is an important ECM protein and has a crucial role in the maintenance of normal morphology, cell adhesion, migration, and wound healing [20]–[21], Thus, FN is postulated to be a key role in the signaling pathways that led to these changes observed in this study. We proposed that the inhibition of complex I by knocking down *GRIM-19* or *NDUFS3* gene led to increased ROS production and the generated ROS then modulate ECM and its related proteins, such as FN, and its receptor, integrin α5, and the interaction between these proteins activates downstream signal pathways, leading to enhanced metastatic properties.

Our study demonstrated that suppression of mitochondrial complex I activity by performing siRNA knockdown of *GRIM-19* or *NDUFS3* gene can affect expression of ECM molecules and its related proteins, promoting cell invasive properties, such as cell adhesion, migration, and invasion, as well as spheroid formation. These observations suggest that impaired mitochondrial complex I may have an active effect on metastasis.

## Materials and Methods

### Cell Culture, Antibodies and Other Reagents

Hela cells were cultured in Eagle’s minimum essential medium (EMEM) (Sigma, St Louis, Il, USA) supplemented with 10% fetal bovine serum (FBS) (Gibco). The cell lines MDA-MB-231, Hs578T, ZR-75-1, BT549, MDA-MB-453, MDB-MA-468, SK-BR-3 and BT-474 were obtained from Dr Zeng’s laboratory at Institute of Molecular and Cell Biology, Singapore [22]. BT-549 cells and ZR-75-1 were cultured in RPMI supplemented with 10% FBS from Gibco-BRL and MDA-MB-231, Hs578T, MDA-MB-453, MDB-MA-468, SK-BR-3, BT-474 were maintained in Dulbecco’s modified Eagle’s medium (DMEM) with 10% FBS. Antibodies against GRIM-19, integrin β1 were purchased from Calbiochem (Darmstadt, Germany). NDUFS3 and FN antibodies were from Molecular Probes (Eugene, OR, USA) and Abcam (Cambridge, MA, USA), respectively. Antibodies against N-cadherin, integrin α5, desmoplakin and vimentin were from Santa Cruz Biotechnology (Santa Cruz, CA, US). Mucin 1 and HIF-1α antibodies were also purchased from Cell Signaling Technology (Beverly, USA).

### Knockdown Genes with siRNA

Knockdown of *GRIM-19* or *NDUFS3* by siRNA were performed in HeLa cells using pSUPER.neo (OligoEngine, Seattle, WA, USA) following the manufacturer’s instructions. Stable transfected cell lines were established after selection with neomycin. The detailed methods were described previously [6].

### Western Blot Analysis

Cells were lysed using RIPA buffer (150 mM NaCl, 50 nM Tris-HCl, pH 7.2, 1% deoxycholic acid, 1% Triton X-100, 0.1% sodium dodecyl sulfate (SDS), 0.25 mM EDTA) with proteinase inhibitors (Roche, Basel, Switzerland) after washing with cold phosphate-buffered saline (PBS). Cell lysates containing equal amounts of total proteins from wild type cells or cells transfected with scrambled, *GRIM-19* or *NDUFS3* siRNA were separated by SDS-polyacrylamide gel eletrophoresis (SDS-PAGE) after being boiled in Laemmli buffer and transferred to polyvinylidence difluoride (PVDF) membrane. The blot was blocked in PBS with 0.1% Tween 20 and 1% bovine serum albumin overnight, and then incubated with appropriate primary and second antibodies for 1 hr each, and the bound antibodies were visualized by chemiluminescene (Amersham Bioscience. UK).

### Cell Adhesion Assay

HeLa cells were cultured in 2% FBS for 24 hr and 30 min in serum free medium before detaching with 0.25% trypsin for 1 min. After washing with PBS, 5×10^5^ cells were resuspended in fresh serum free medium and then seeded onto plates coated with FN (20 µg/ml, Sigma) or collagen-coated dishes (Iwaki) and incubated at 37°C with 5% CO_2_. After 30 min incubation, the medium was removed and the plates were washed with PBS to remove the unattached cells. The attached cells were fixed with 3.7% paraformaldehyde and countered using a phase-contrast microscope (Leica DM4000 B Wetzlar, Germany). The cell numbers were obtained from five randomly selected fields with three independent experiments.

### Cell Migration, Invasion Assay

Wound healing and transwell migration assays were performed to assess cell motility. For wound healing, the cells were cultured to confluence and treated with mitomycin C for 2 hours to arrest cell proliferation. A wound track was made using a P200 pipette tip and the old medium and cell debris were removed. The plates were washed with PBS and the cells were grown in fresh medium for further 10 hours. Phase-contrast images of the wound area were taken just after scratching and after 10-hour re-culturing.

Transwell cell migration assay was carried out using 24-well transwell (8-µm pores, Costar Corning, NY, USA) with the underside of filter unit coated with 20 µg/ml of FN. Each 0.5 ml of serum free-medium containing 2×10^5^ cells was applied onto the upper migration chamber. The lower chamber was filled with 1 ml medium with 10%FBS. After incubation for 4 h, non-migrated cells on the upper side of the filter were removed using cotton swabs, and the cells on the underside were stained with 0.4% crystal violet in 10% methanol. The membrane of the filter was then cut and mounted on slides. The images were taken under microscope (Leica DM4000 B) and the attached cells were counted. For each cell group, the number of migrated cells in four different fields was counted and three independent experiments were performed.

For cell invasion assay, the upper chamber of transwell plate was coated with 1 mg/ml growth factor reduced Matrigel basement membrane matrix (BD Biosciences, USA) in serum free medium. The plates were incubated at 37°C for 4h for gelling the matrigel. Each well was then washed with warm serum free medium before plating 1×0^5^ cells in 100 µl of medium containing 2% charcoal filtered FBS. The bottom chamber was filled with 600 µl of medium with 10% FBS which acted as the chemoattractant. The cells were then incubated for 24 hr and followed further steps as in the transwell migration assay.

### Cell Proliferation Assay

Cells (5×10^5^) were seeded in 3.5 cm dishes and cultured at 37°C with 5% CO2. At 24 h, 48, 72, 96 h, 120 h and 144 h, respectively, the cells were harvested and counted with a hemocytometer. Each assay was carried out at least three independent experiments in duplicate.

### Spheroid Formation

Hela cells were harvested using 0.25% trypsin and washed with PBS. 2×10^5^ cells were seeded in 1 ml medium in each well of 6-well plates coated with 1% agarose and grown at 37°C under humid atmosphere with 5% CO_2_. The spheroid was observed and image was taken at different time course. After taking photographs, the cells were collected and lysed with RIPA buffer and used to determine various protein concentrations.

### Flow Cytometric Analysis of ROS

Fluorescent dye, 2′,7′-Dichlorodihydrofluorescein diacetate (H_2_DCFH-DA) (molecular Probes, Oregon) was used to determine the intracellular ROS levels. Cells were harvested and washed with PBS and then exposed to 5 µM H_2_DCFH-DA at 37°C for 1 hour. ROS levels were analyzed by flow cytometry using Becton Dickinson’s FACScan machine.

### Measurement of Complex I Activity

Cells were harvested and mitochondria were isolated according to a protocol as described previously [6]. Complex I activity was measured using spectrophotometric method using NADH as substrate, which was described previously [23]. Briefly, 25 µl of sonicated mitochondrial homogenates were added into 975 µl of pre-warmed reaction system (0.1 M Tris-HCl pH 7.0, 0.3 mM NADH, 0.1 mM Coenzyme Q1, 1 mM KCN and 2 mM NaN3). The reaction mixture was immediately transferred to a pre-warmed (30°C) quartz cuvette. The absorbance of reaction mixture was measured using spectrophotometer at every 20 sec for 5 min at 340 nm. For measurement of the rotenone insensitive NADH oxidation activity, 5 µl of 0.5 mM rotenone was added to the reaction mixture. The enzymatic activity was measured as same as described above.

### Immunofluorescence

Cells were grown on glass cover slips overnight. The cells were washed with PBS and fixed with 4% paraformaldehyde solution for 20 min and permeabilized with PBS containing 0.2% Triton X-100 for 20 min, and blocked with PBS containing 10% FBS for 30 min. Cells were then incubated with appropriate primary antibodies diluted in PBS with 1% BSA and then detected using Cy2/Cy3-conjugated secondary antibodies. The cover slips were mounted on to the glass slides and examined with confocal microscope (Olympus Fluoview Inverted Confocal Microscope).

### Statistic Analysis

All data are presented as mean ± SD. We assessed differences between groups by two-tailed non-paired Student’s *t* test using the Graphpad Prism statistical software. A p value less than 0.05 was considered as statistical significance.
