# Correction: Suppression of Mitochondrial Complex I Influences Cell Metastatic Properties

**DOI:** 10.1371/journal.pone.0097052

**Published:** 2014-04-30

**Authors:** The PLOS ONE Staff

There is an error in one result of this article. Knockdown of GRIM-19 did not inhibit cell proliferation as reported, but increased it. Please note the following changes to the text:

The third paragraph of the Results section should read:

In addition to the cell-matrix interaction, we also examined cell-cell adhesion by spheroid formation assay in three-dimensional culture. We observed that round and tight spheroids were formed at 48 hr in G19 and p30 cells, while no obvious spheroid was observed in WT and SC at the same time point (Figure 2F). In order to exclude the possibility that the increased migration and invasion in knockdown cells were due to increased cell proliferation, we used mitomycin C to treat the cell lines for 2 hours to arrest cell proliferation before wound healing and transwell migration assays (Please see the method part of cell migration, invasion assay in page 6). In addition, we examined the cell proliferation rate and found that the proliferation rates in the G19 or p30 cells were significantly increased (p<0.01) (Figure 2G). These observations demonstrated that the suppression of GRIM-19 or NDUFS3 in HeLa cells promoted cell adhesion, migration, invasion and spheroid formation, and increased the cell proliferation.

The second sentence in the third paragraph of the Discussion section should read:

Our results showed that knockdown of GRIM-19 or NDUFS3 induced EMT, enhanced cell adhesion, migration, invasion, spheroid formation, and increased cell proliferation.

This change does not alter the main conclusions of this article.

## References

[1] He X, Zhou A, Lu H, Chen Y, Huang G, et al (2013) Suppression of Mitochondrial Complex I Influences Cell Metastatic Properties. PLoS ONE 8(4): e61677 doi:10.1371/journal.pone.0061677 23630608 10.1371/journal.pone.0061677 PMC3632579

