# Correction: Suppression of Mitochondrial Complex I Influences Cell Metastatic Properties

**DOI:** 10.1371/journal.pone.0303435

**Published:** 2024-05-02

**Authors:** Xuelian He, Aifen Zhou, Hao Lu, Yong Chen, Guochang Huang, Xin Yue, Peiwei Zhao, Yanxiang Wu

After the publication and correction of this article [1, 2], additional concerns were noted about Fig 2A, 2D and 2G. Specifically:

- In Fig 2A, the WT and SC (scrambled control) panels appear to report overlapping image data.
- The quantitative migration data for G19 are missing from the bar graph in Fig 2D.
- In Fig 2G, the growth curves were labelled incorrectly.

These errors are corrected in the updated version of Fig 2 provided here. The underlying image data for Fig 2 are provided with this notice in S1 File, and data for other figures are available upon request from the corresponding author. The corresponding author noted that the quantitative data underlying Fig 2 graphs and the western blot data for Integrin β1, HIF-1α, Mucin 1, and Desmoplakin in Fig 3A are no longer available.

The authors apologize for the errors in the published article.

**Fig 2:**
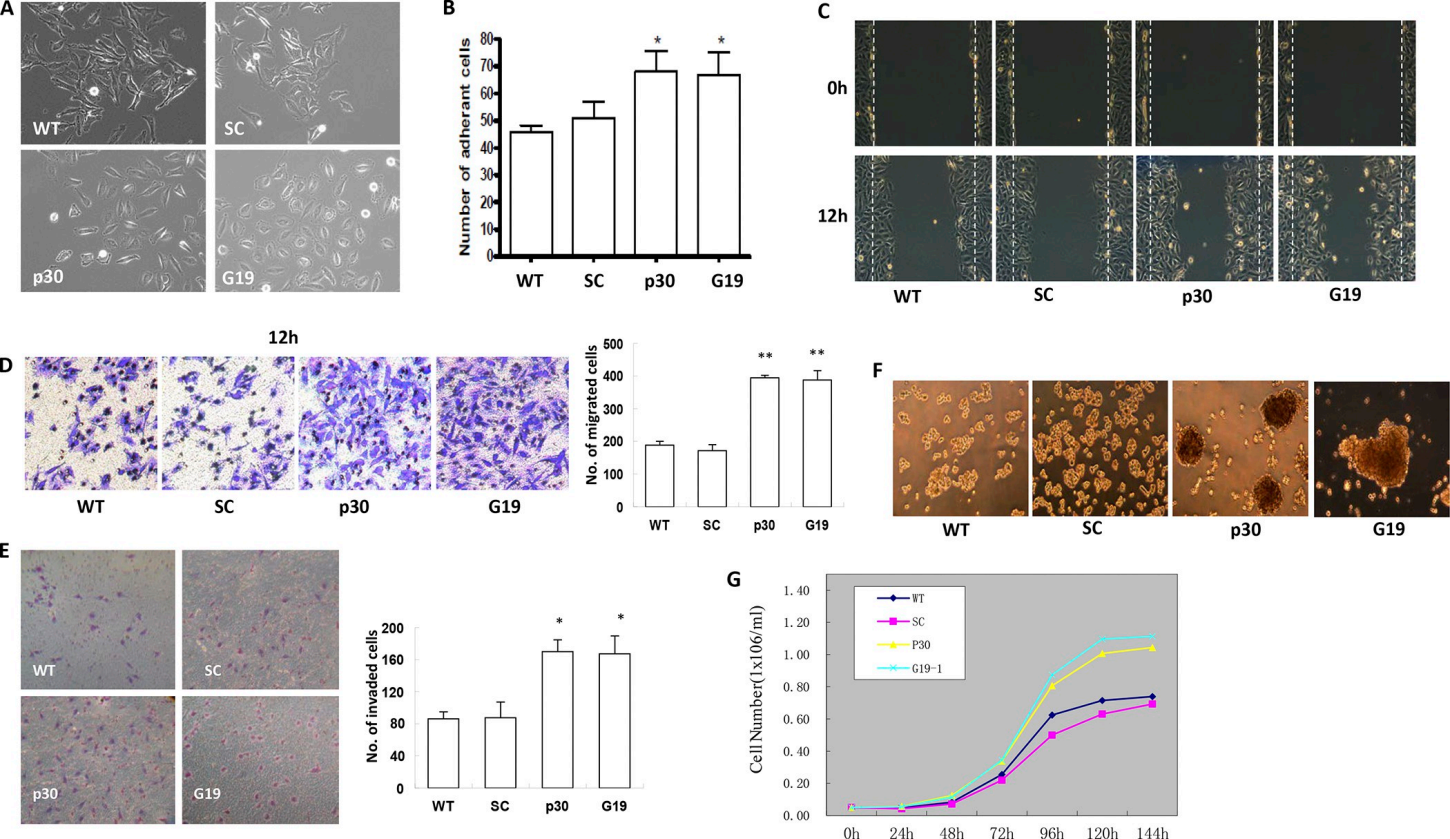
Knockdown of GRIM-19 and NDUFS3 in Cells Induced EMT-Like Phenotypes and Inhibited Cell Proliferation. Cells were seeded onto plates coated with FN and cultured for indicated times at 37°C for one day in CO2 incubator. After washing using PBS and fixing using 3.7% paraformaldehyde, the cell morphology was examined under a phase-contrast microscope (A). Serum-starved Hela cells (5×105) were seeded onto collagen-coated plates and cultured for 30 min. After washing with serum free media, the attached cells were counted and compared (B). Migration assay was assessed using wound healing and transwell methods, respectively. The healing of wound was imaged at 12 hr in wound-healing assay (C) and the migrated through the filter membrane of transwell were stained with 0.4% crystal violet and imaged (D). Cell invasion was tested by using Matrigel-coated transwell filters and the cells invaded through Matrigel and filter were imaged (E). Spheroid formation was evaluated by seeding 5000 cells/ml of each cell lines into 1% agarose gel-coated plates. The cells were cultured at normal culture conditions and the spheroid formation was monitored at appropriate time points (F). Knockdown GRIM-19 and NDUFS3 reduce cell proliferation in normal culture conditions (G). Asterisks indicate a p-value of ≤0.05 (*) or ≤0.01(**) as determined by Student’s T-test.

## Supporting information

S1 File Raw image data to support Fig 2. (ZIP)
