# Supplementary material for: Correction: Suppression of Mitochondrial Complex I Influences Cell Metastatic Properties
Source: PLoS One. 2024 May 2;19(5):e0303435. doi: 10.1371/journal.pone.0303435 (PMC11065242; doi:10.1371/journal.pone.0303435)
Supplement: S1 File — (ZIP) [file pone.0303435.s001.zip › Figure 2E original 2.pptx]

## Slide 1
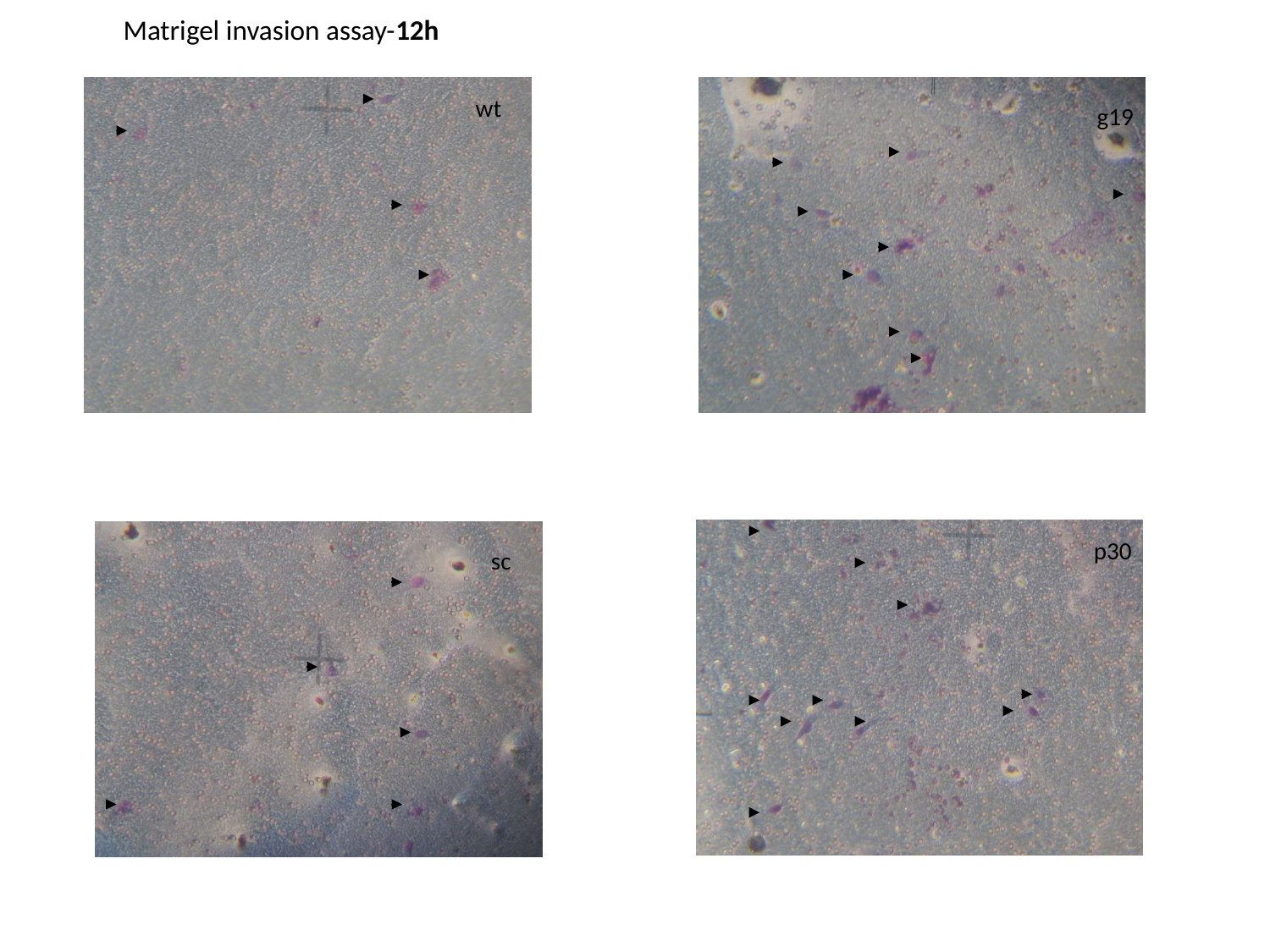

Matrigel invasion assay-12h
wt
g19
p30
sc

## Slide 2
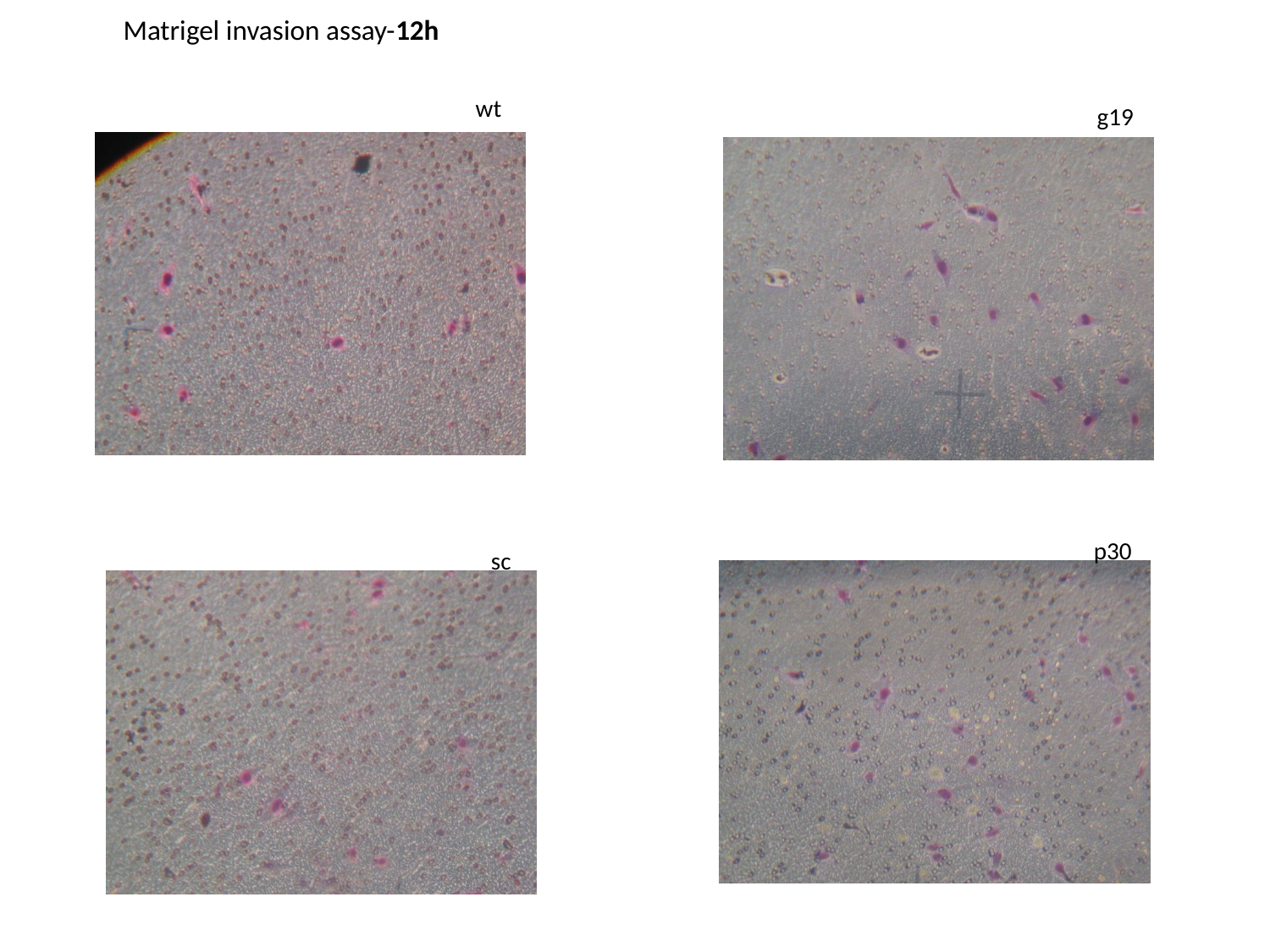

Matrigel invasion assay-12h
wt
g19
p30
sc

## Slide 3
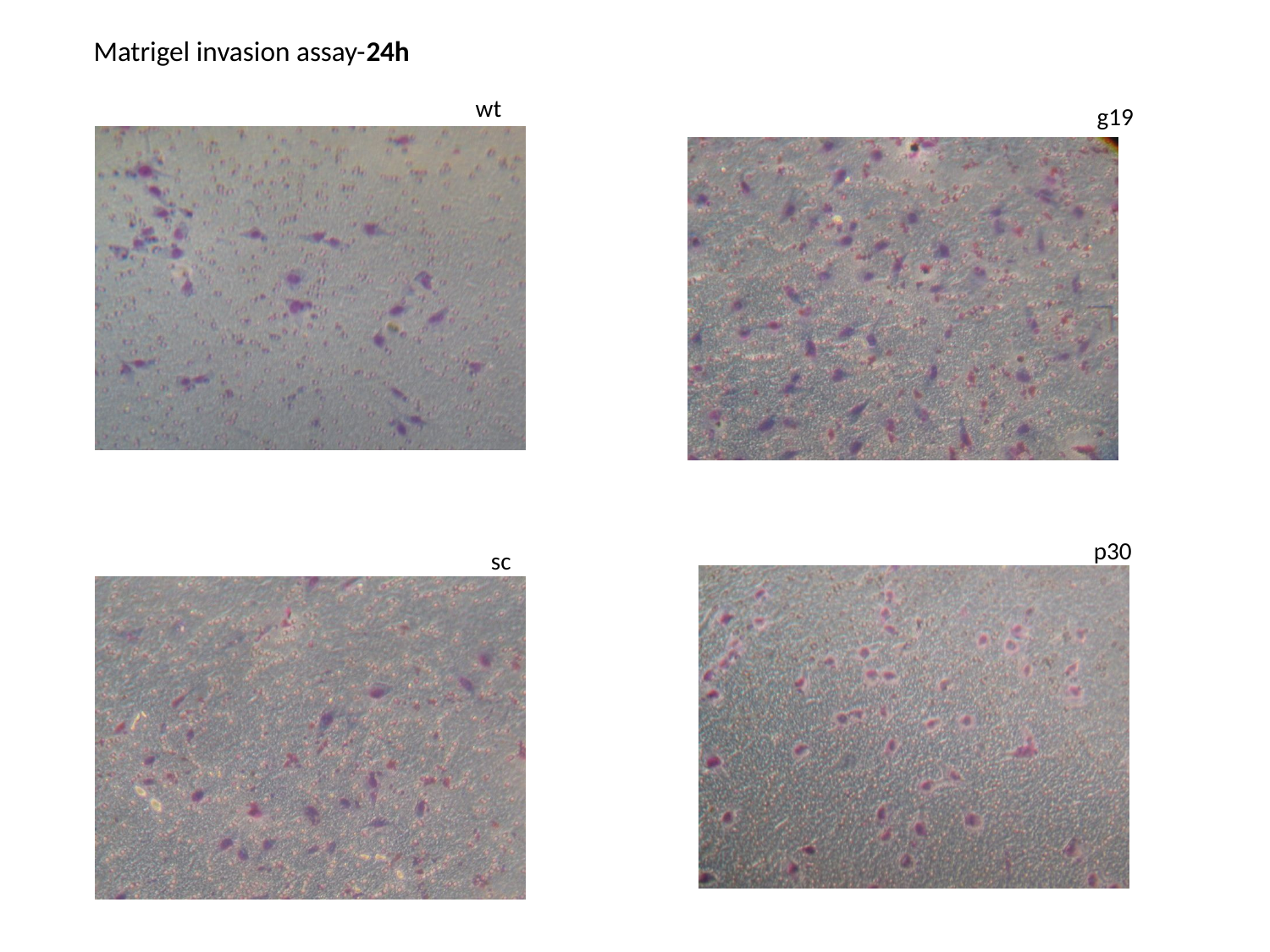

Matrigel invasion assay-24h
wt
g19
p30
sc
