# Supplementary figures and images for: Correction: Suppression of Mitochondrial Complex I Influences Cell Metastatic Properties
Source: PLoS One. 2024 May 2;19(5):e0303435. doi: 10.1371/journal.pone.0303435 (PMC11065242; doi:10.1371/journal.pone.0303435)

## Slide 1
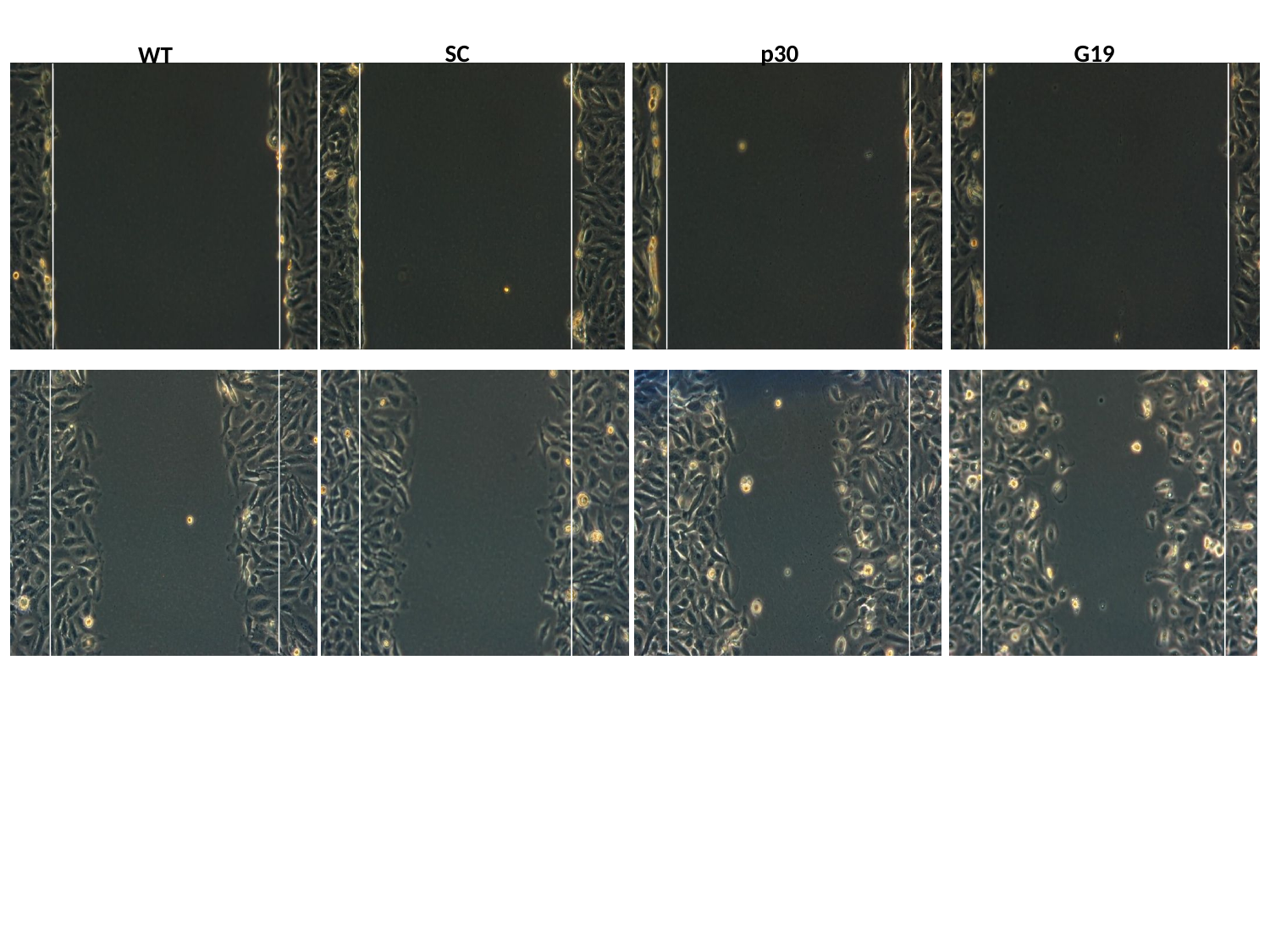

SC
p30
G19
WT

## Slide 2
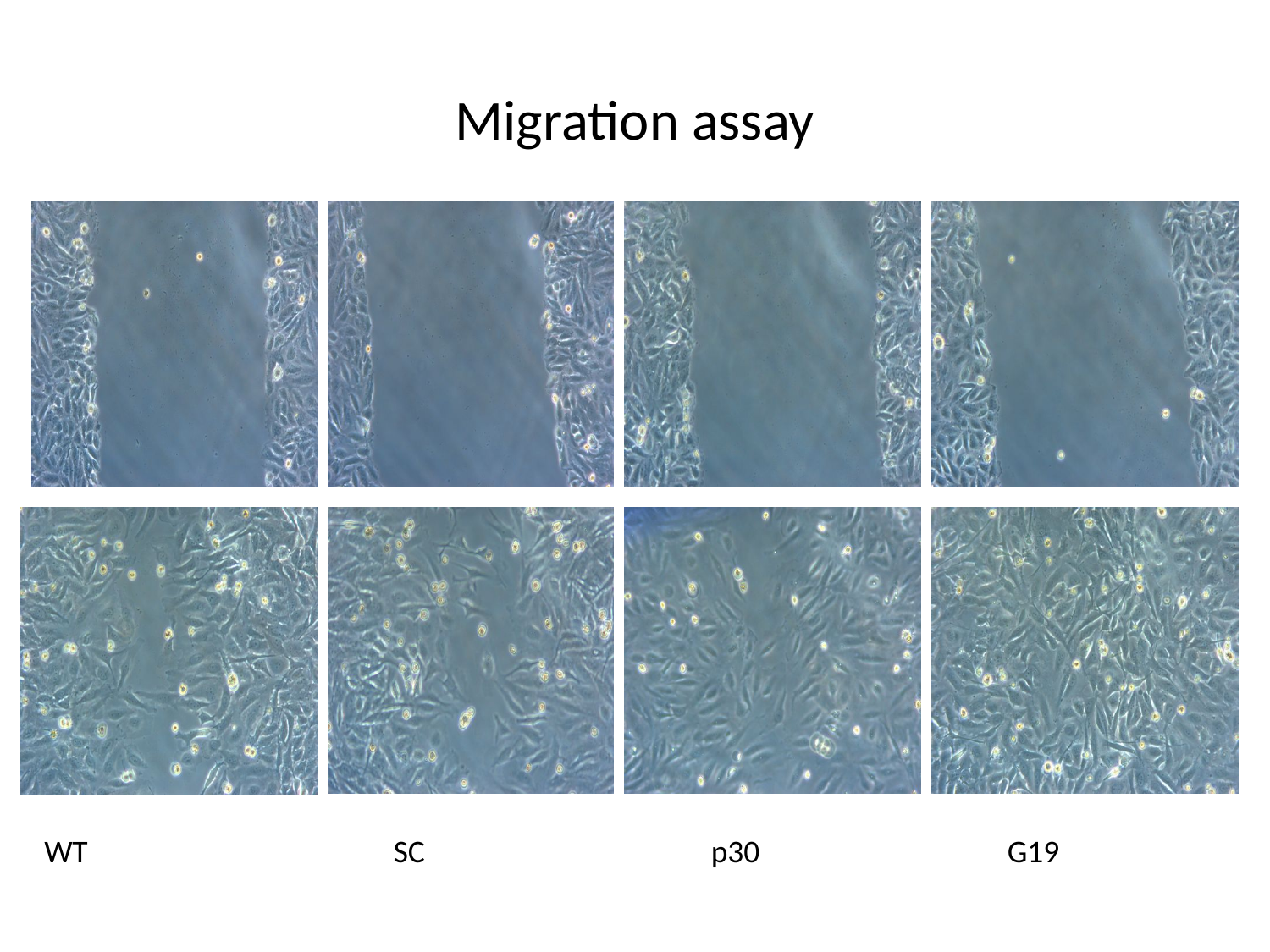

# Migration assay
WT
SC
p30
G19

Supplement: S1 File — (ZIP) [file pone.0303435.s001.zip › Figure 2C original.pptx]

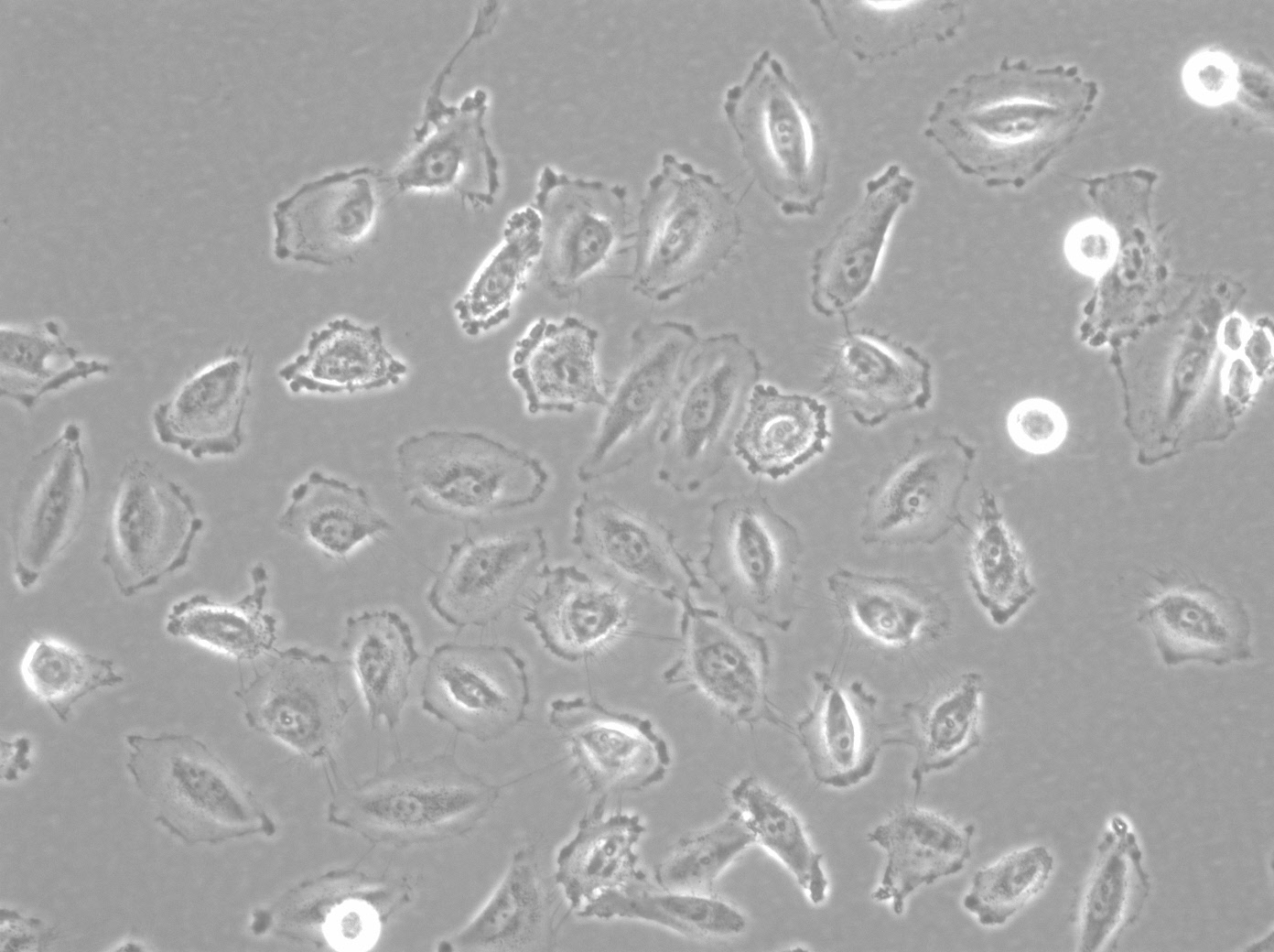

Supplement: S1 File — (ZIP) [file pone.0303435.s001.zip › File 1 - images for Figure 2A morphology/G19.jpg]

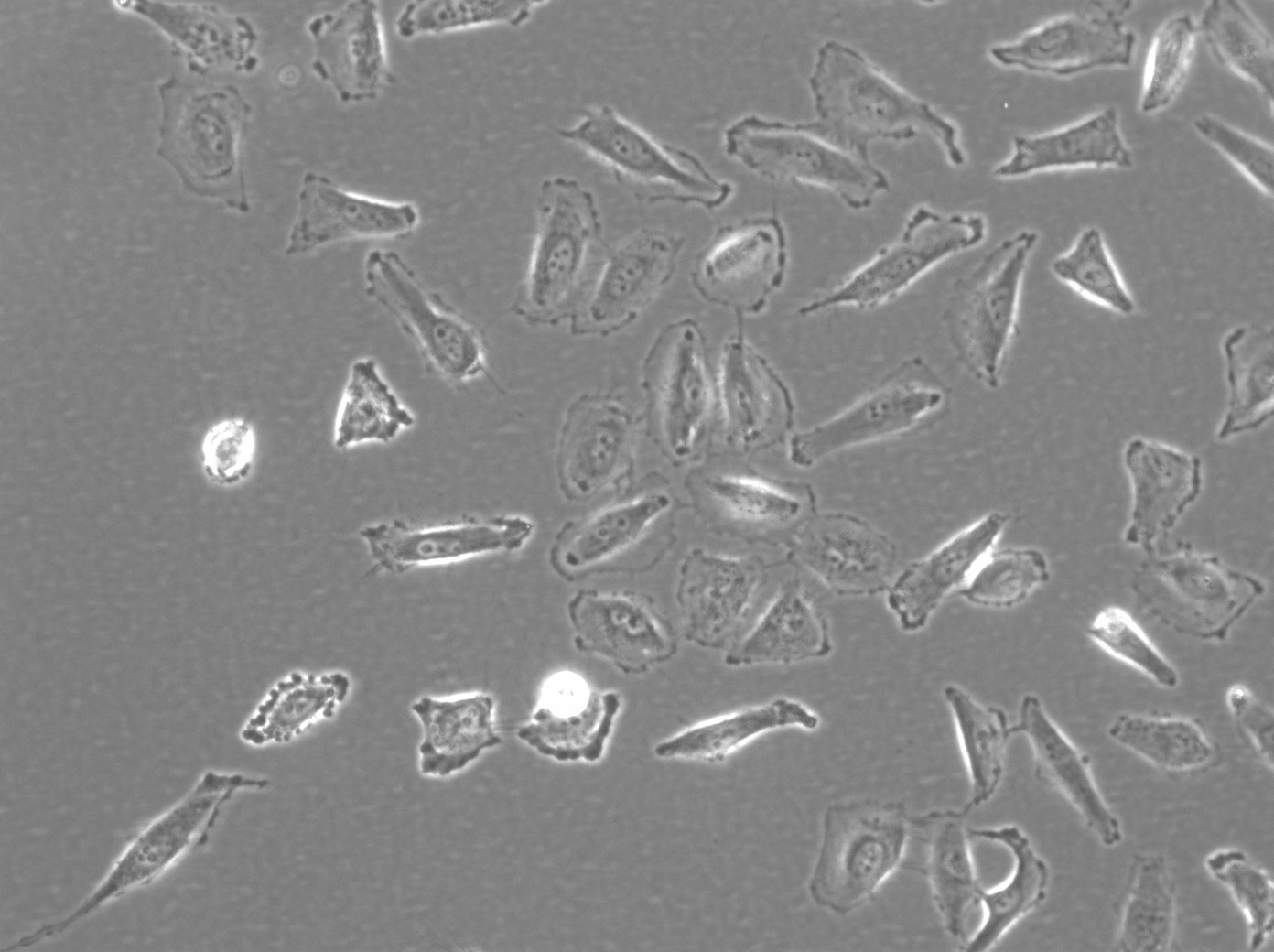

Supplement: S1 File — (ZIP) [file pone.0303435.s001.zip › File 1 - images for Figure 2A morphology/P30.jpg]

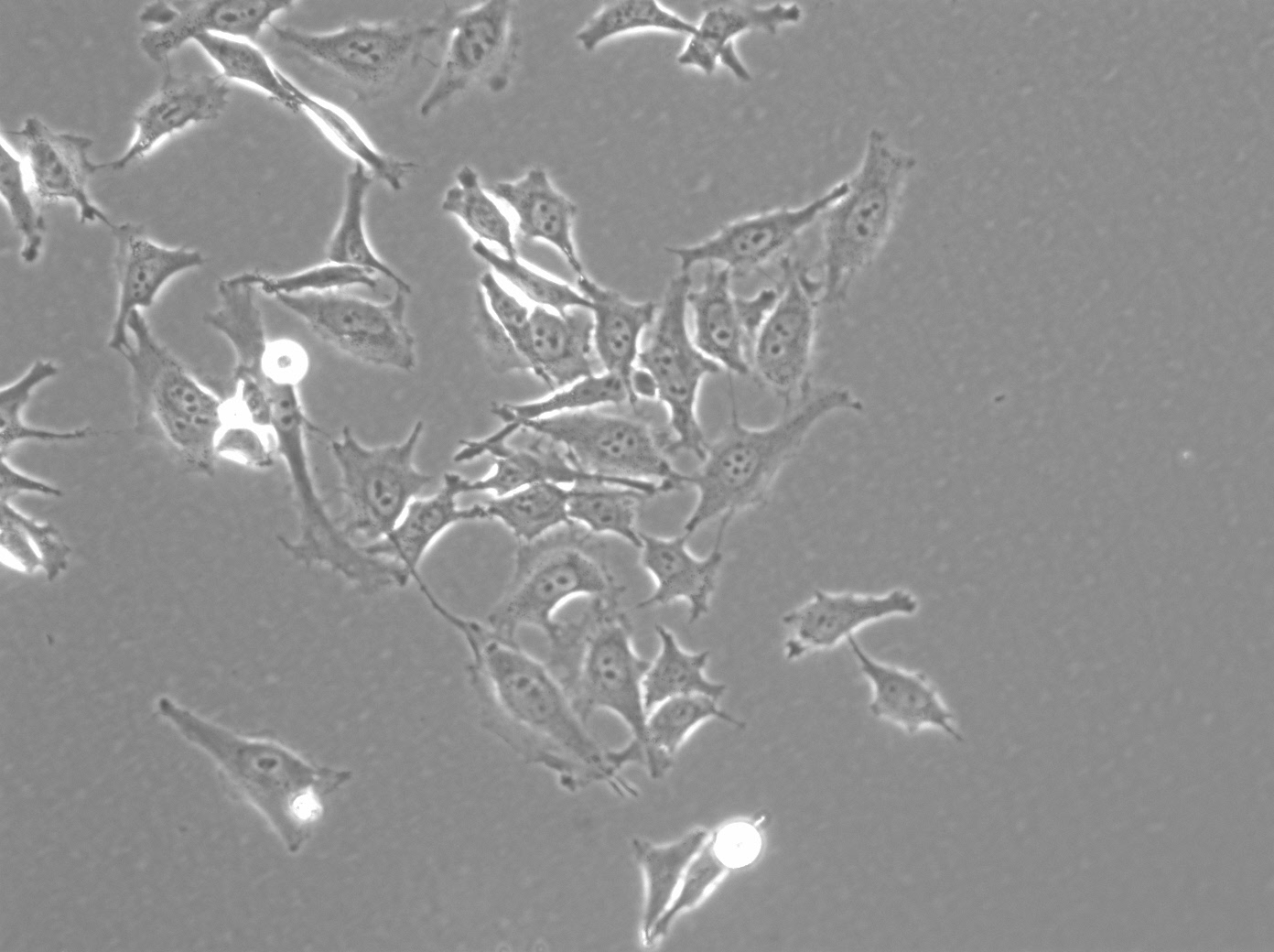

Supplement: S1 File — (ZIP) [file pone.0303435.s001.zip › File 1 - images for Figure 2A morphology/SC.jpg]

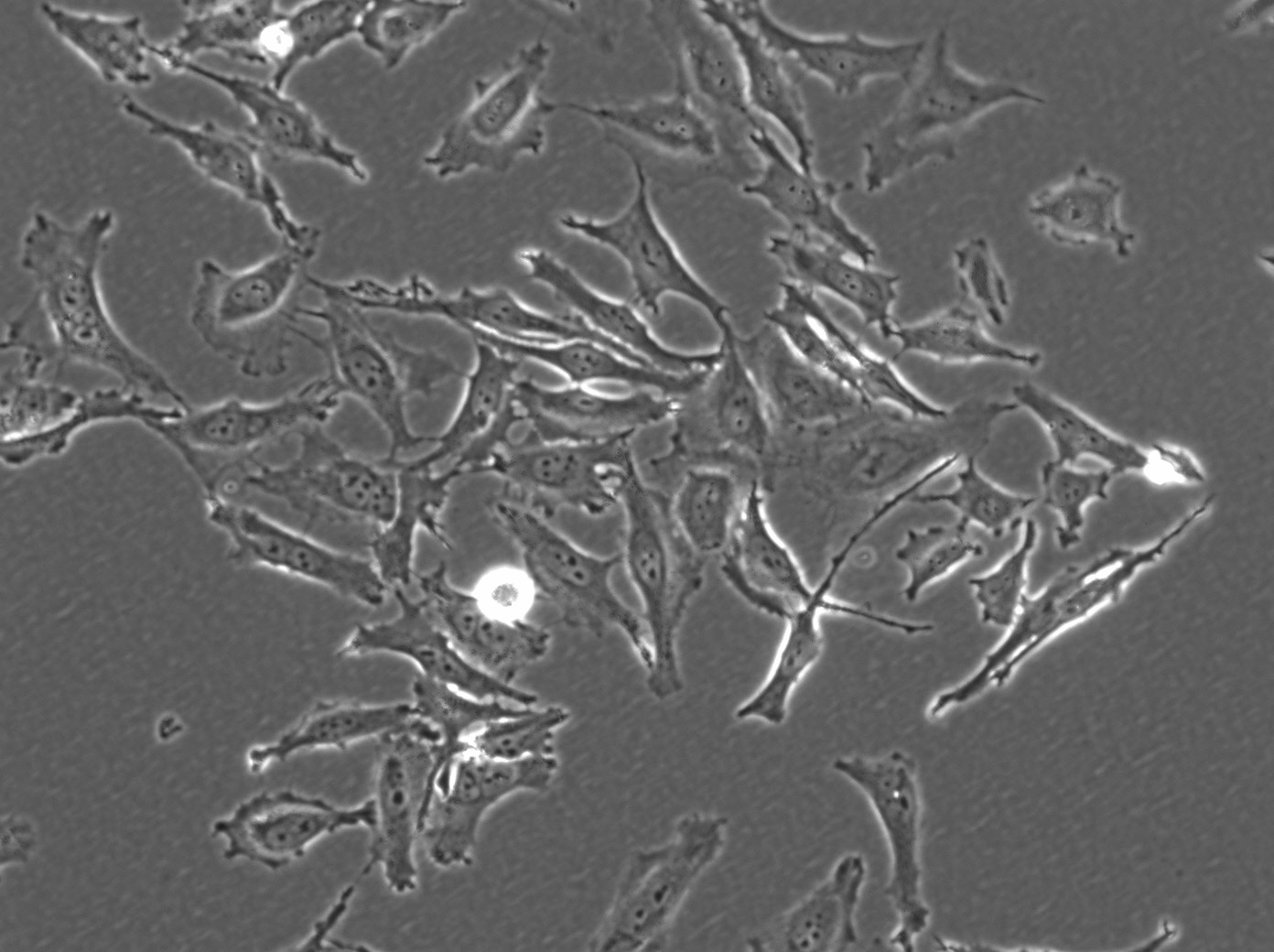

Supplement: S1 File — (ZIP) [file pone.0303435.s001.zip › File 1 - images for Figure 2A morphology/WT.jpg]

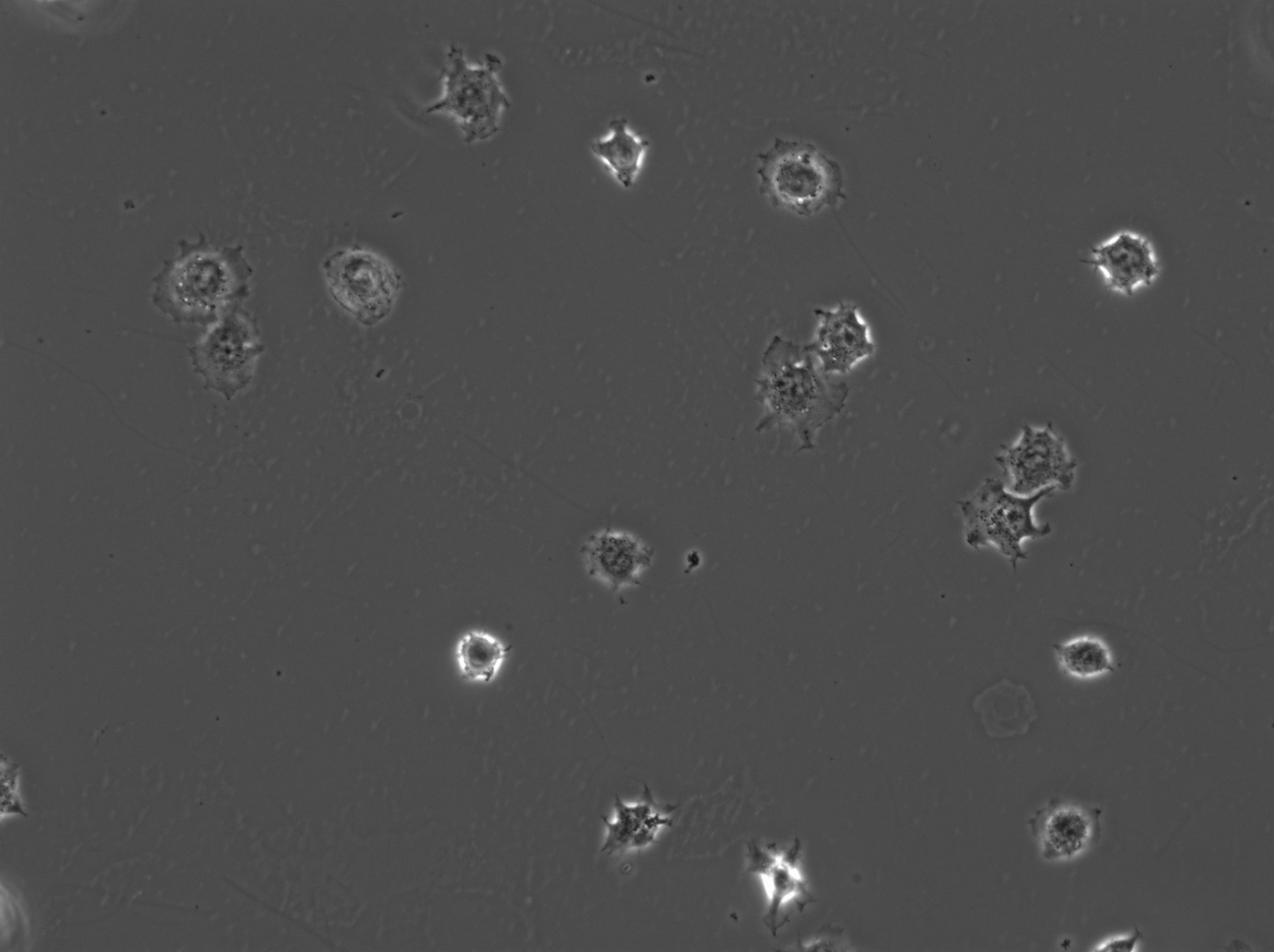

Supplement: S1 File — (ZIP) [file pone.0303435.s001.zip › File 2--Images for Figure 2B Hela cell adhesion assay/image1.png]

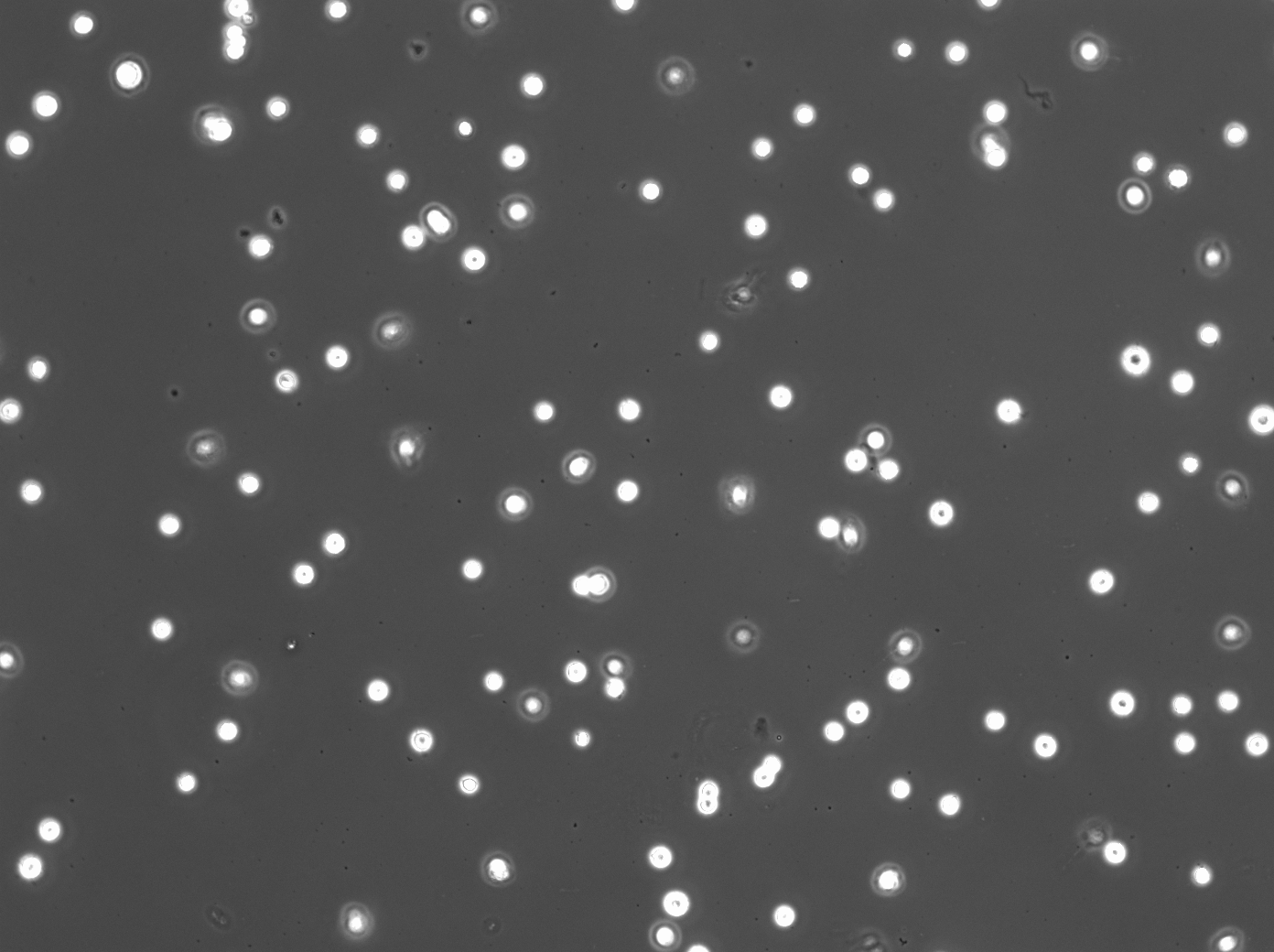

Supplement: S1 File — (ZIP) [file pone.0303435.s001.zip › File 2--Images for Figure 2B Hela cell adhesion assay/image10.png]

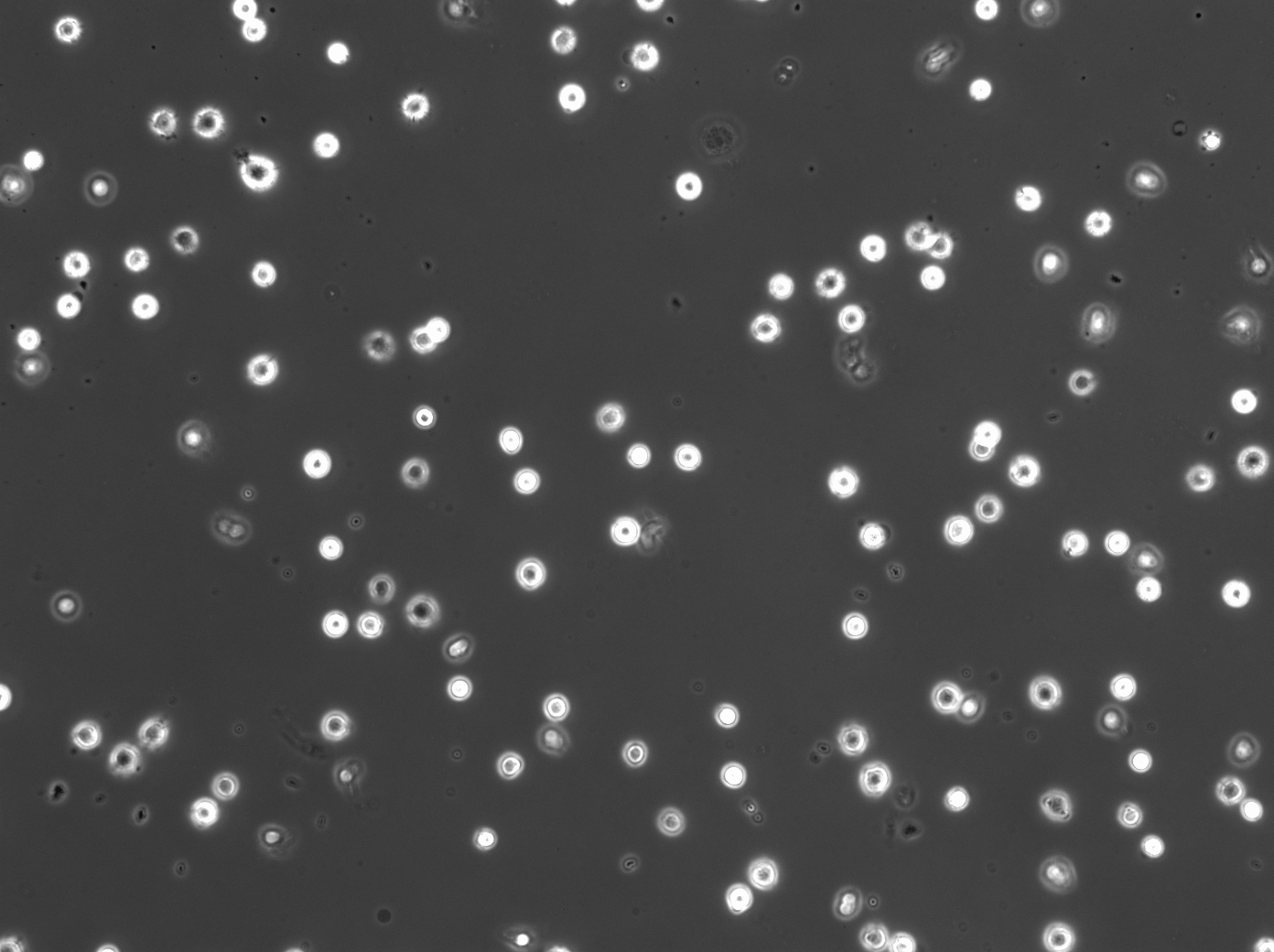

Supplement: S1 File — (ZIP) [file pone.0303435.s001.zip › File 2--Images for Figure 2B Hela cell adhesion assay/image11.png]

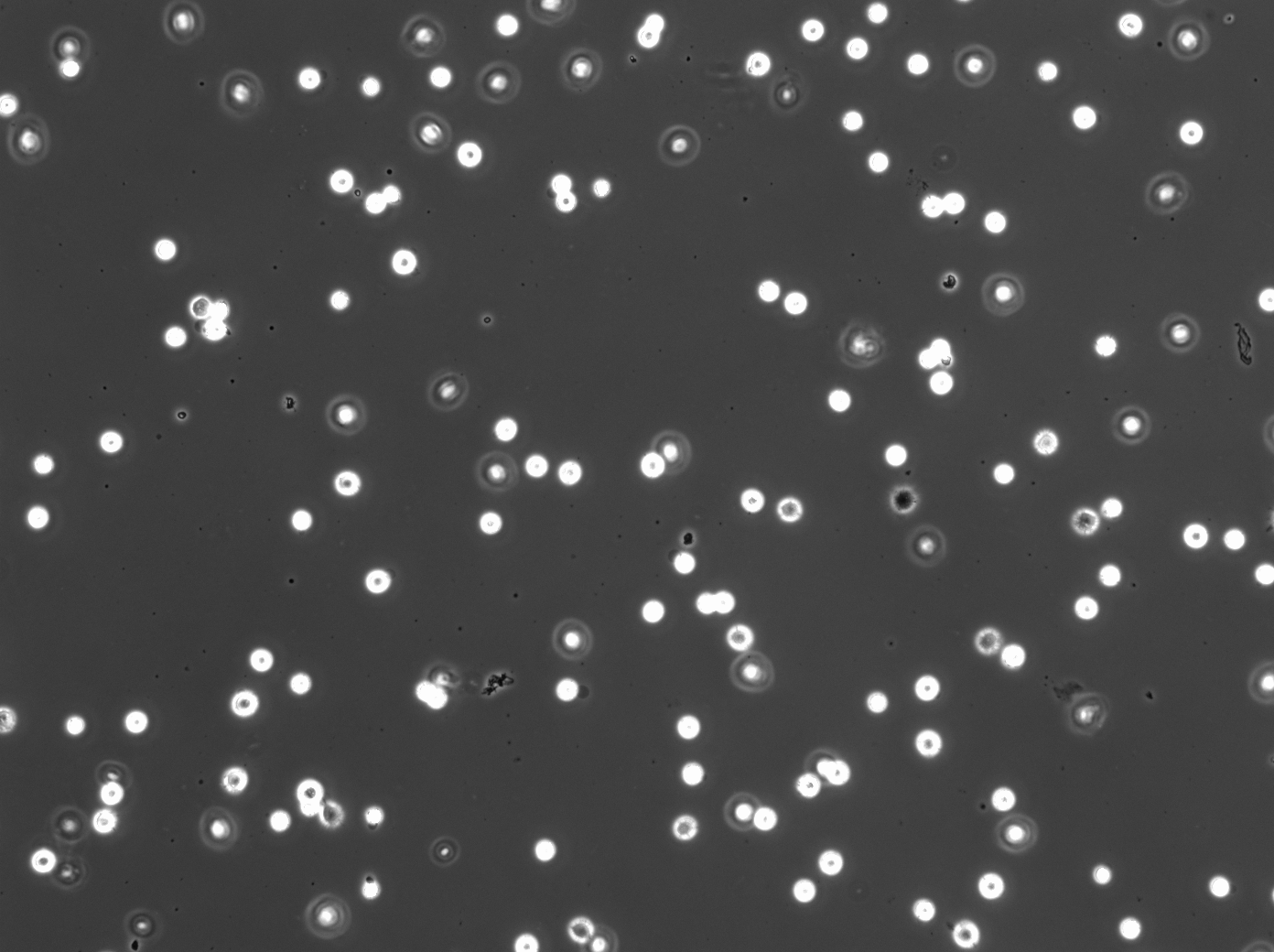

Supplement: S1 File — (ZIP) [file pone.0303435.s001.zip › File 2--Images for Figure 2B Hela cell adhesion assay/image12.png]

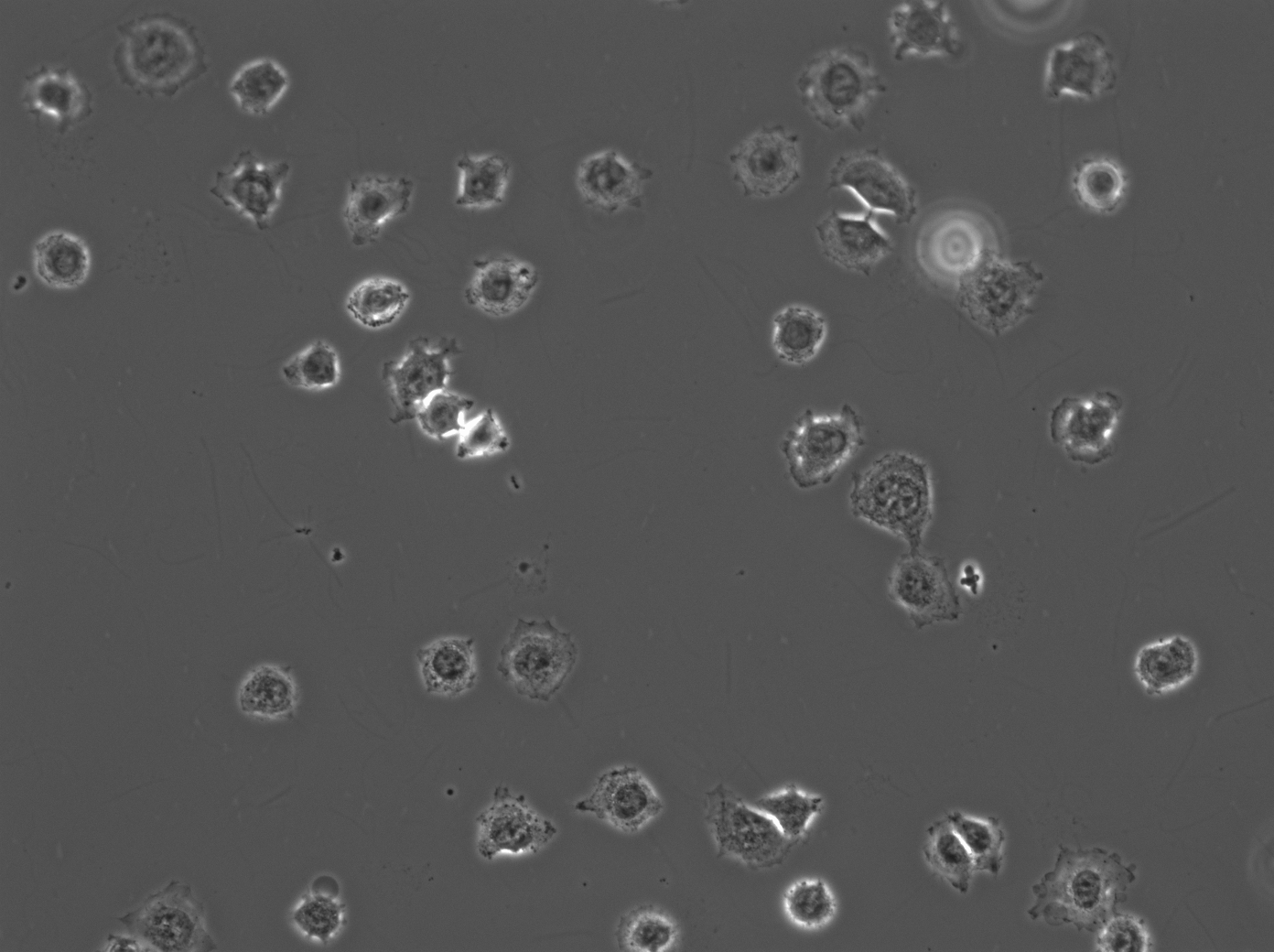

Supplement: S1 File — (ZIP) [file pone.0303435.s001.zip › File 2--Images for Figure 2B Hela cell adhesion assay/image2.png]

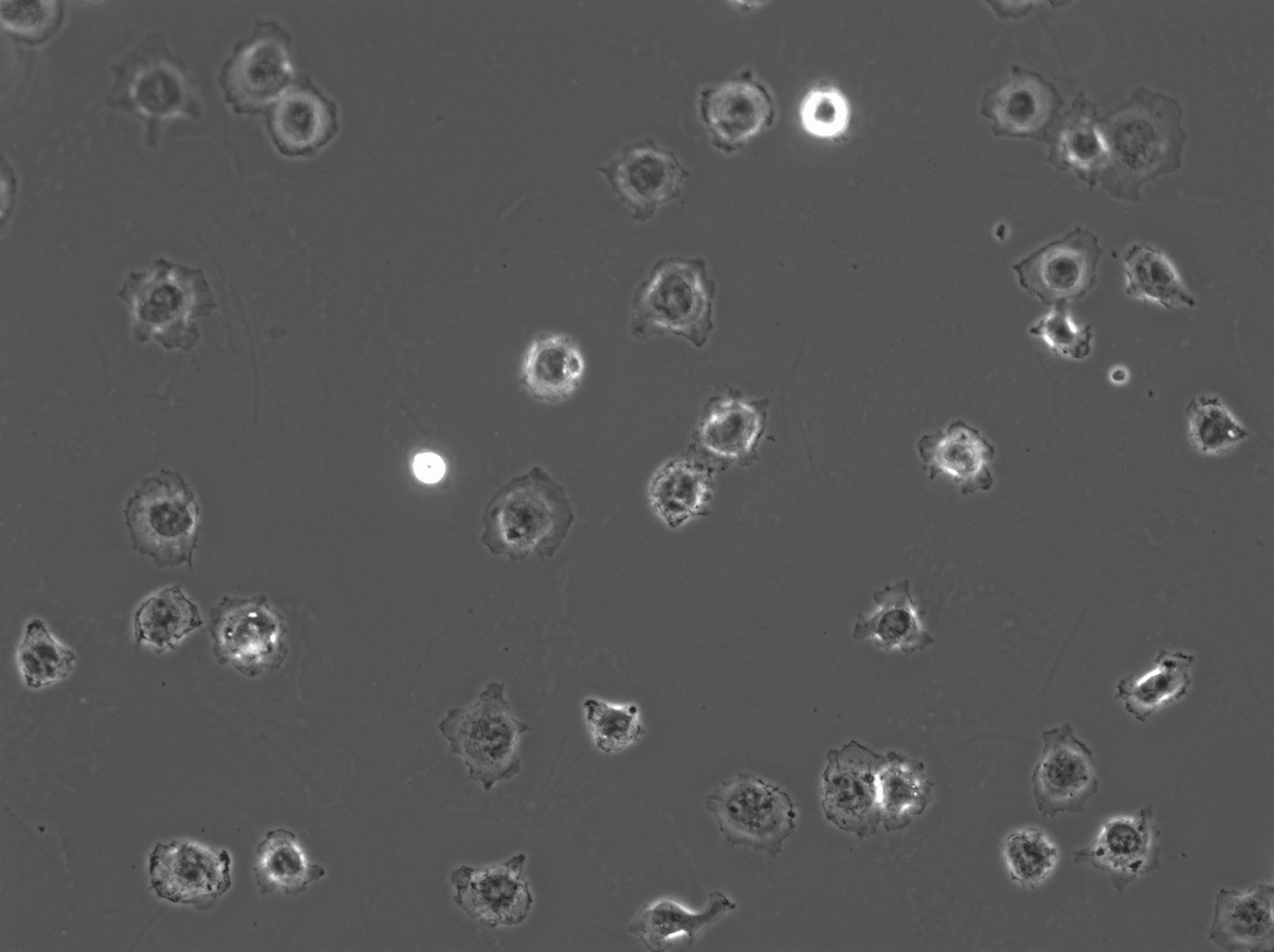

Supplement: S1 File — (ZIP) [file pone.0303435.s001.zip › File 2--Images for Figure 2B Hela cell adhesion assay/image3.png]

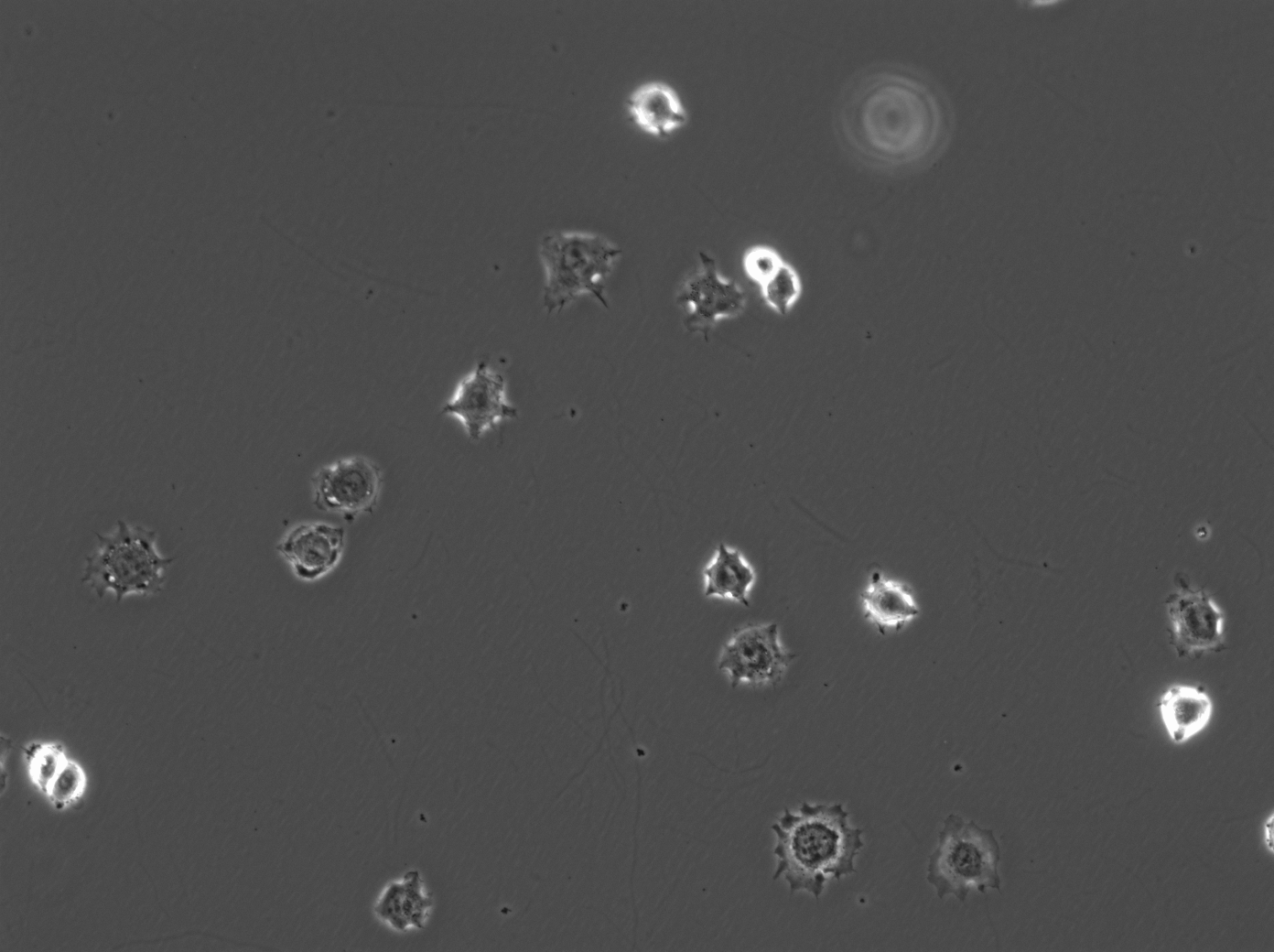

Supplement: S1 File — (ZIP) [file pone.0303435.s001.zip › File 2--Images for Figure 2B Hela cell adhesion assay/image4.png]

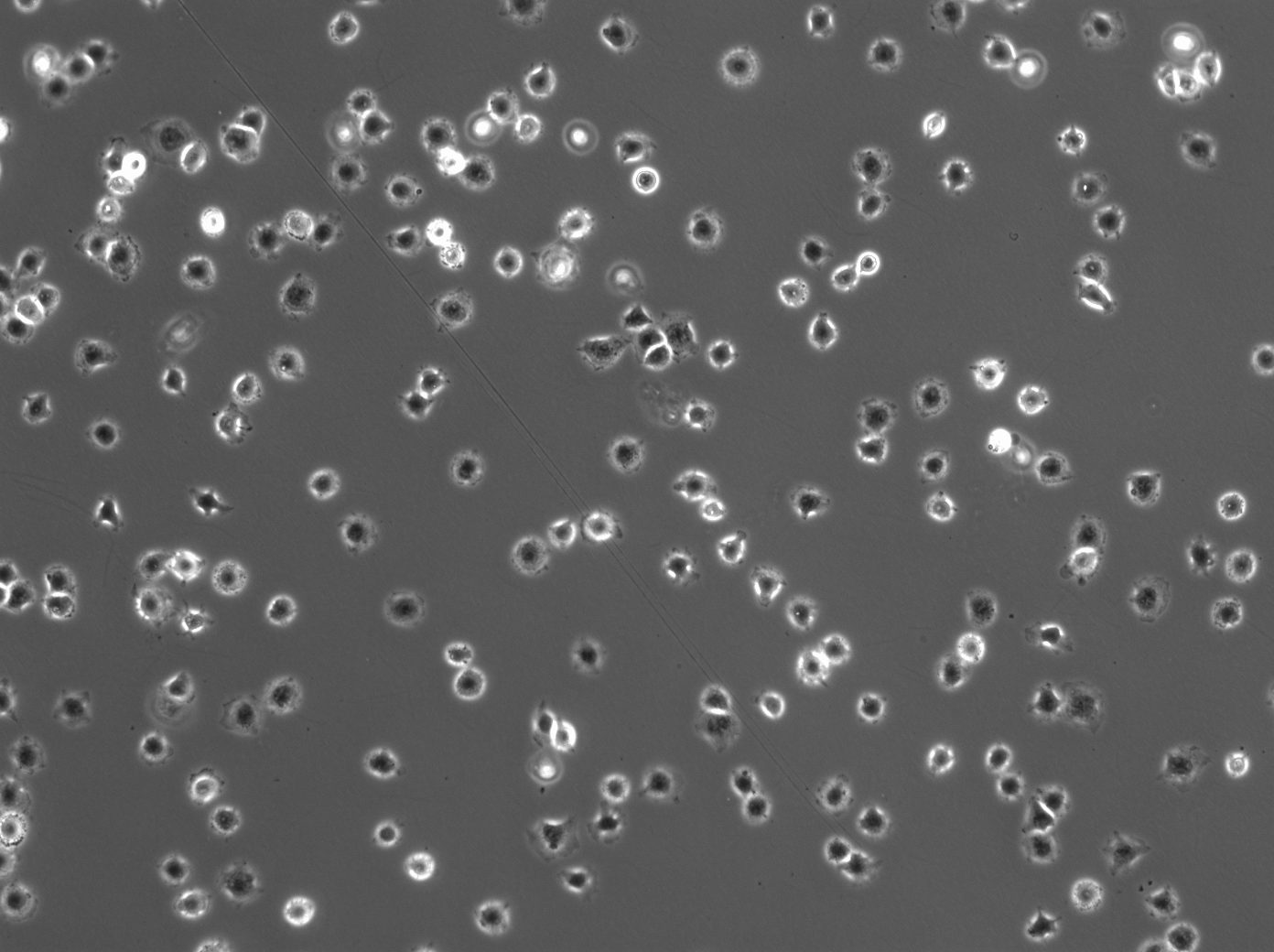

Supplement: S1 File — (ZIP) [file pone.0303435.s001.zip › File 2--Images for Figure 2B Hela cell adhesion assay/image5.png]

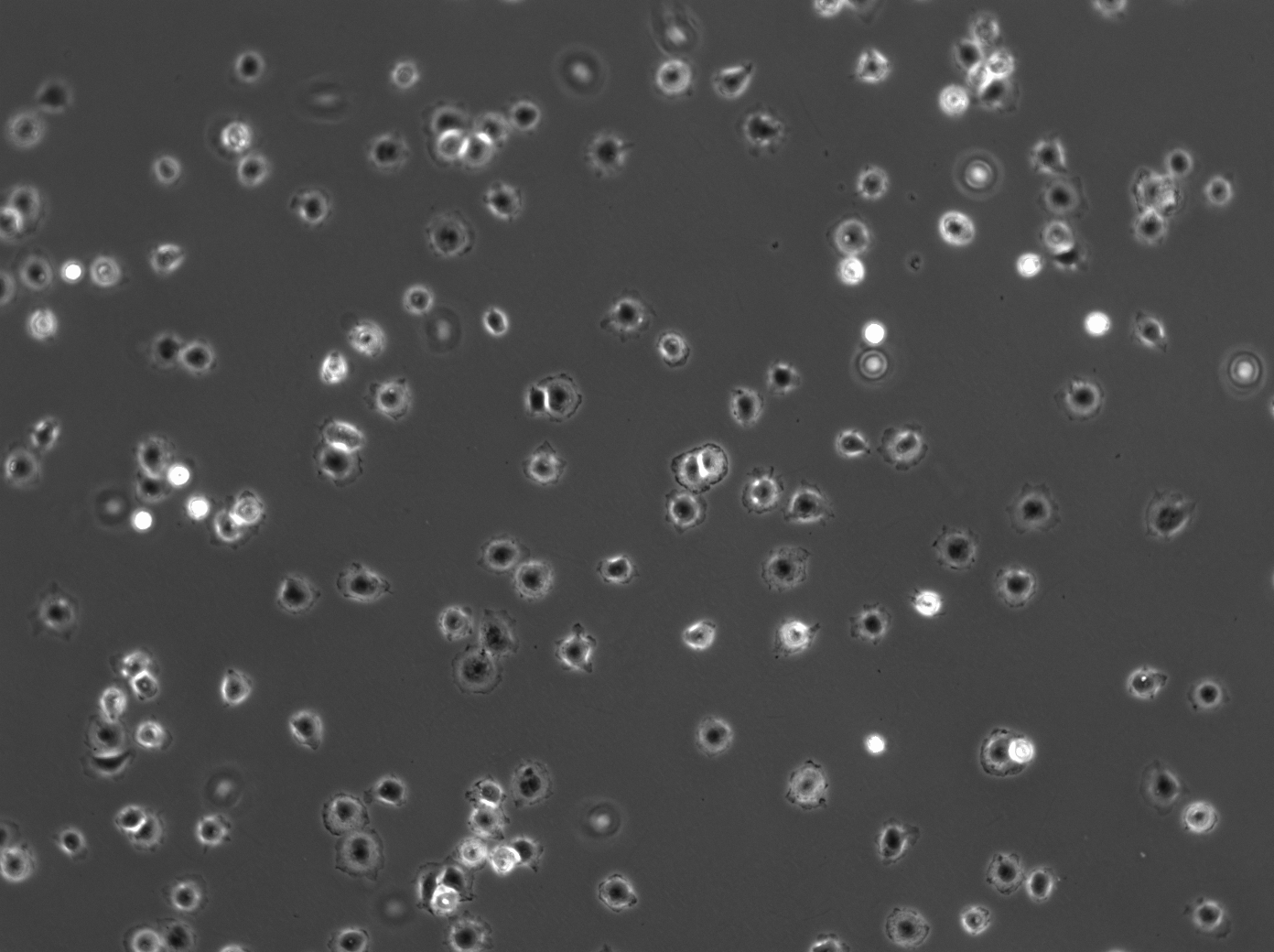

Supplement: S1 File — (ZIP) [file pone.0303435.s001.zip › File 2--Images for Figure 2B Hela cell adhesion assay/image6.png]

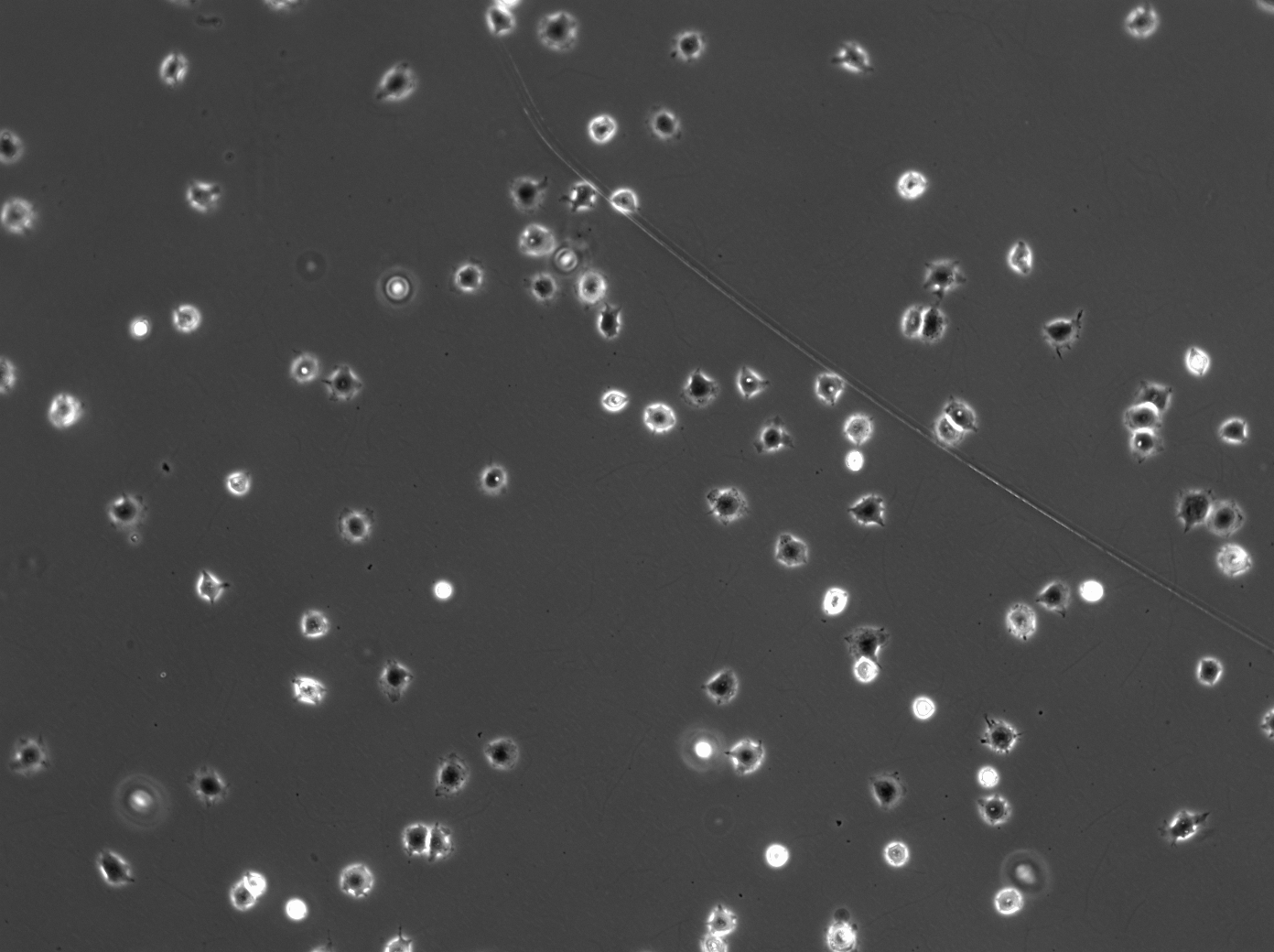

Supplement: S1 File — (ZIP) [file pone.0303435.s001.zip › File 2--Images for Figure 2B Hela cell adhesion assay/image7.png]

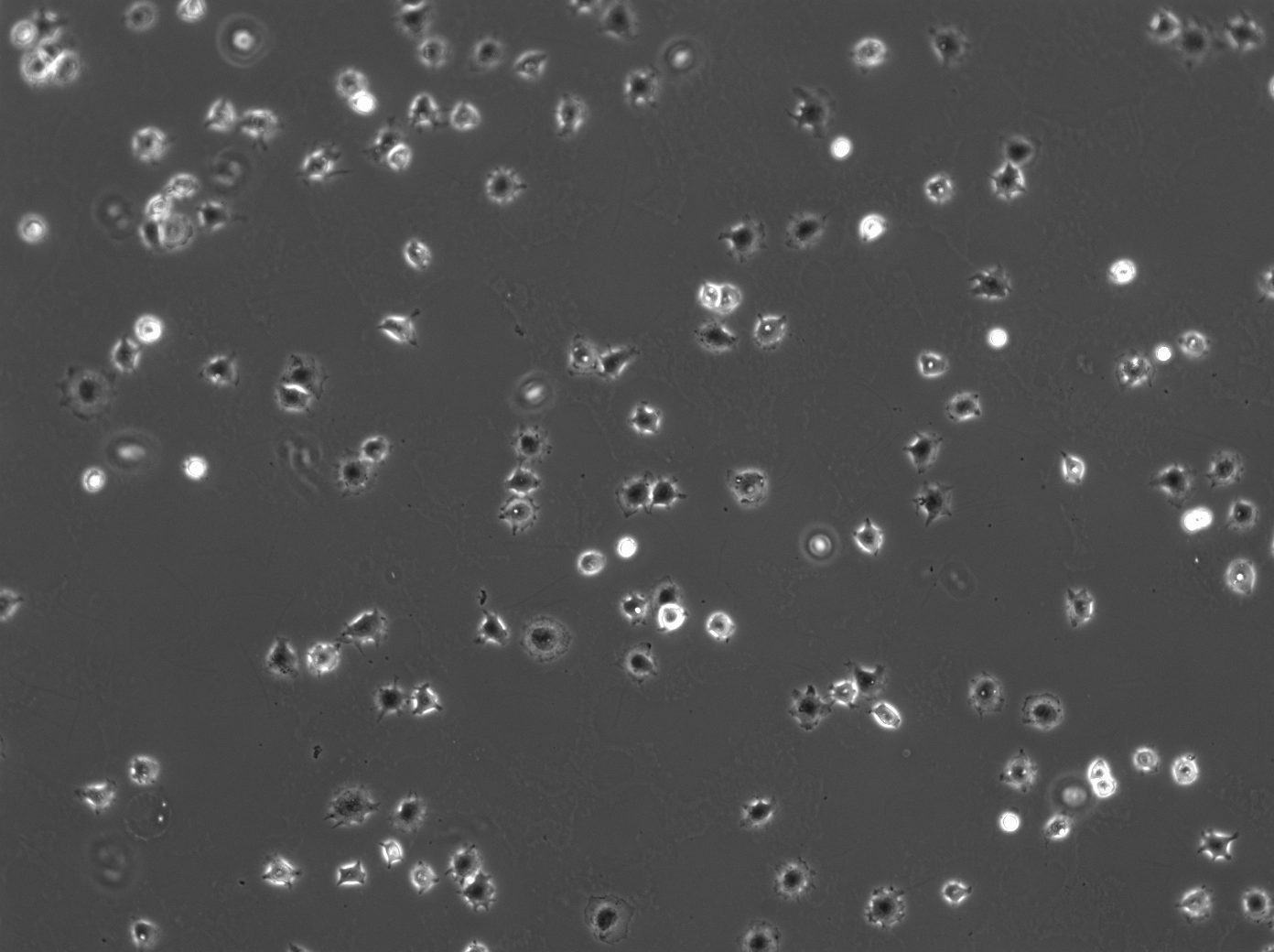

Supplement: S1 File — (ZIP) [file pone.0303435.s001.zip › File 2--Images for Figure 2B Hela cell adhesion assay/image8.png]

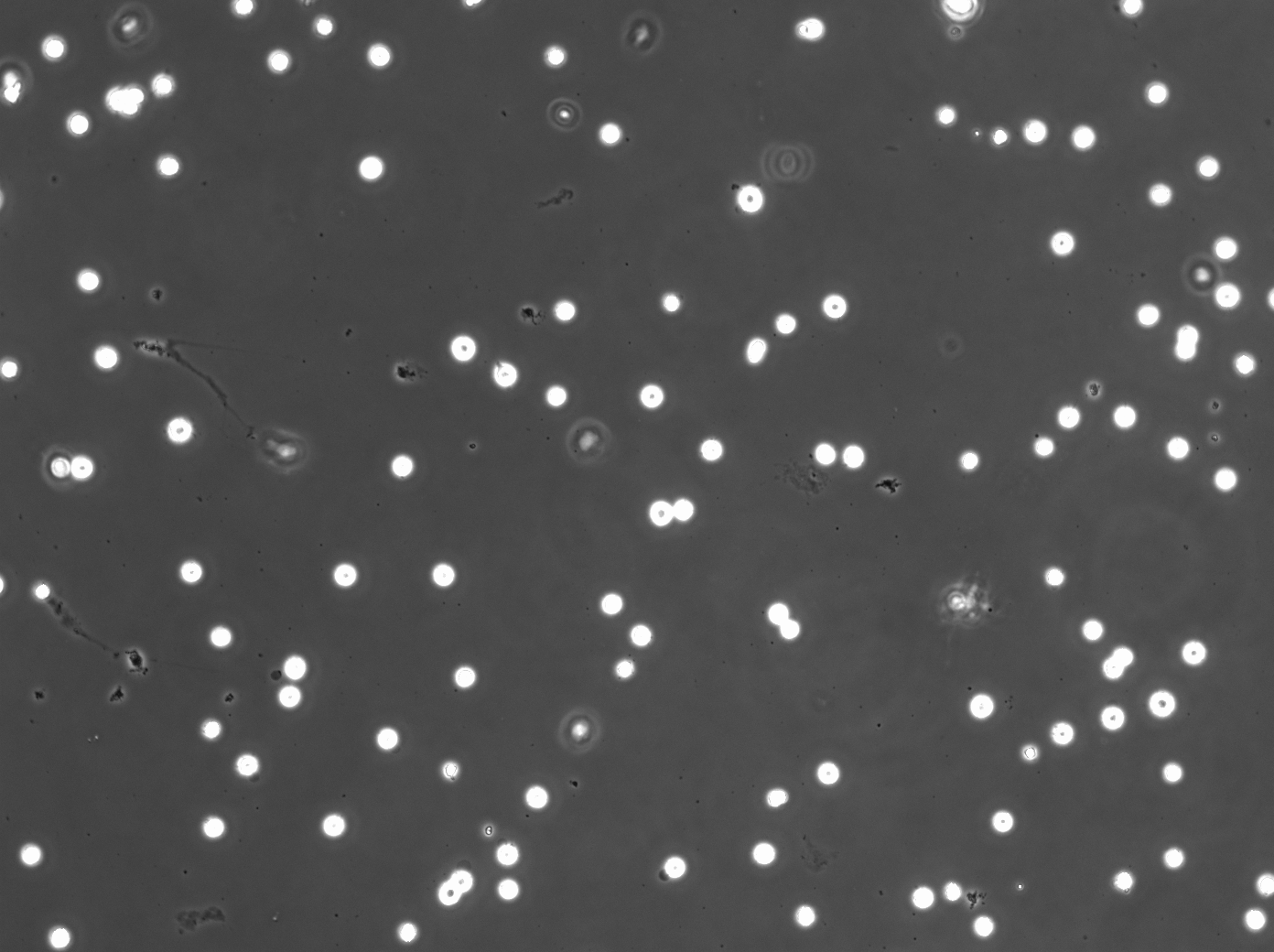

Supplement: S1 File — (ZIP) [file pone.0303435.s001.zip › File 2--Images for Figure 2B Hela cell adhesion assay/image9.png]

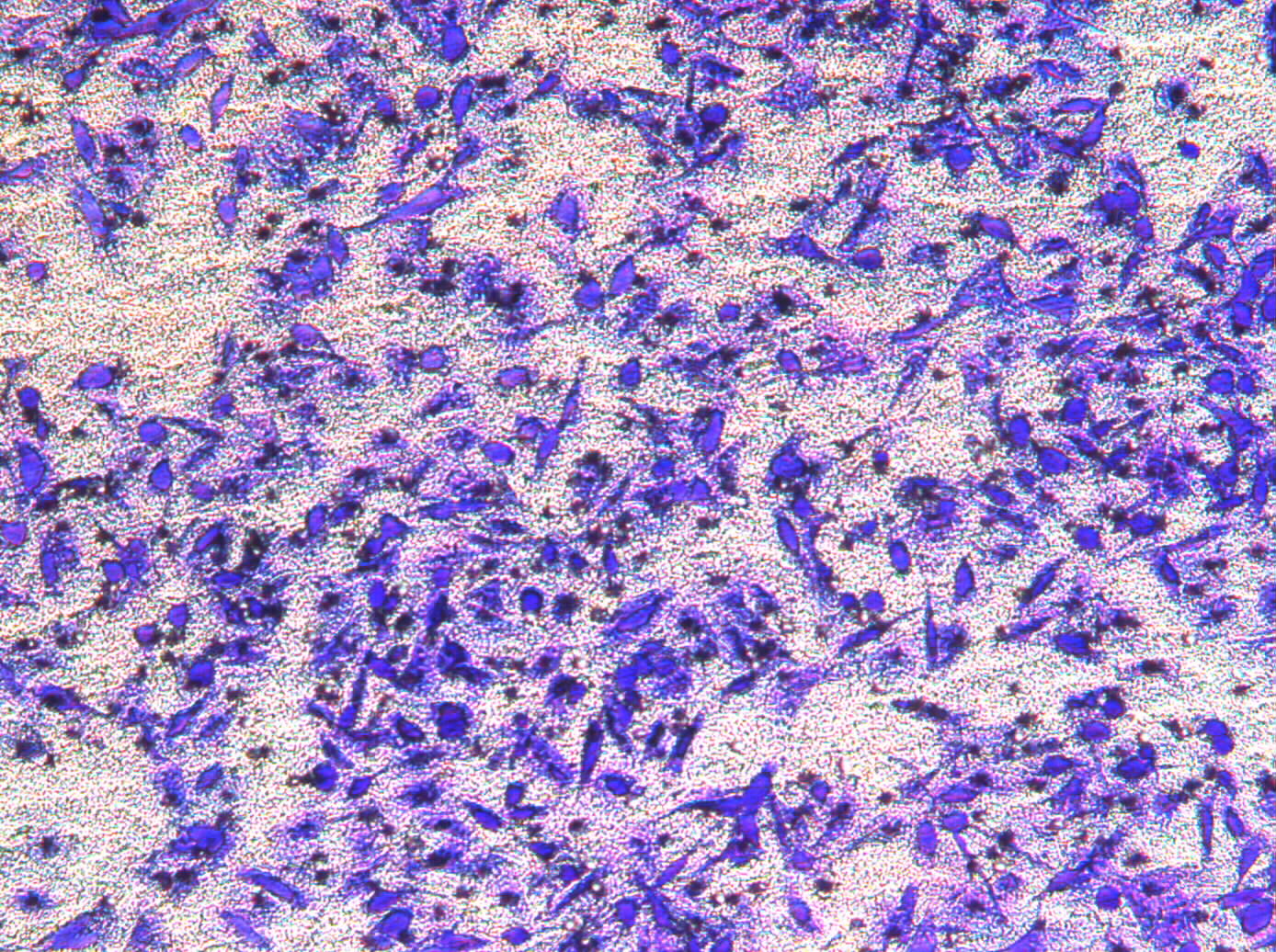

Supplement: S1 File — (ZIP) [file pone.0303435.s001.zip › File 3 - images for Figure 2D transwell/G19 -1.jpg]

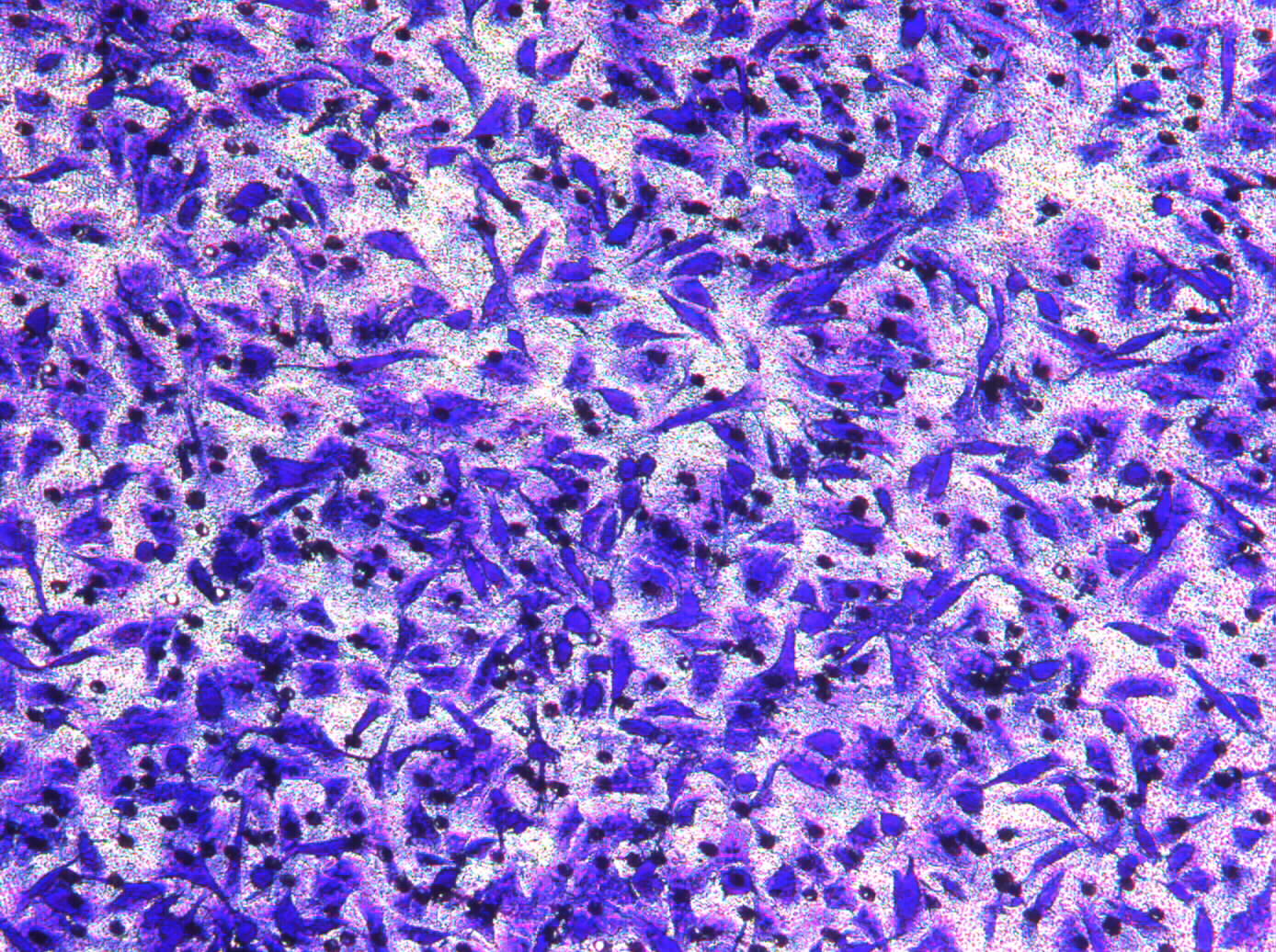

Supplement: S1 File — (ZIP) [file pone.0303435.s001.zip › File 3 - images for Figure 2D transwell/G19-2.jpg]

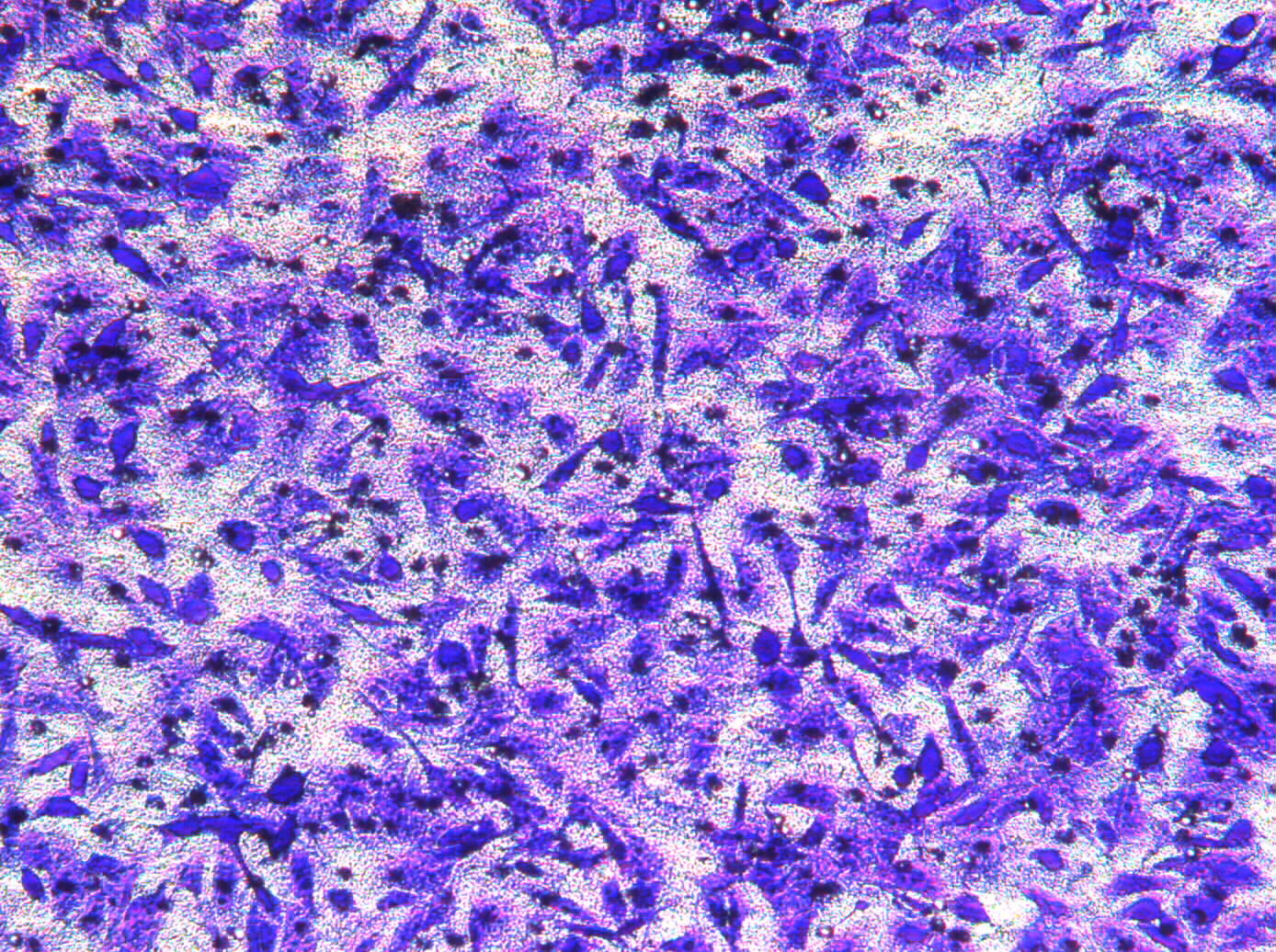

Supplement: S1 File — (ZIP) [file pone.0303435.s001.zip › File 3 - images for Figure 2D transwell/G19-3.jpg]

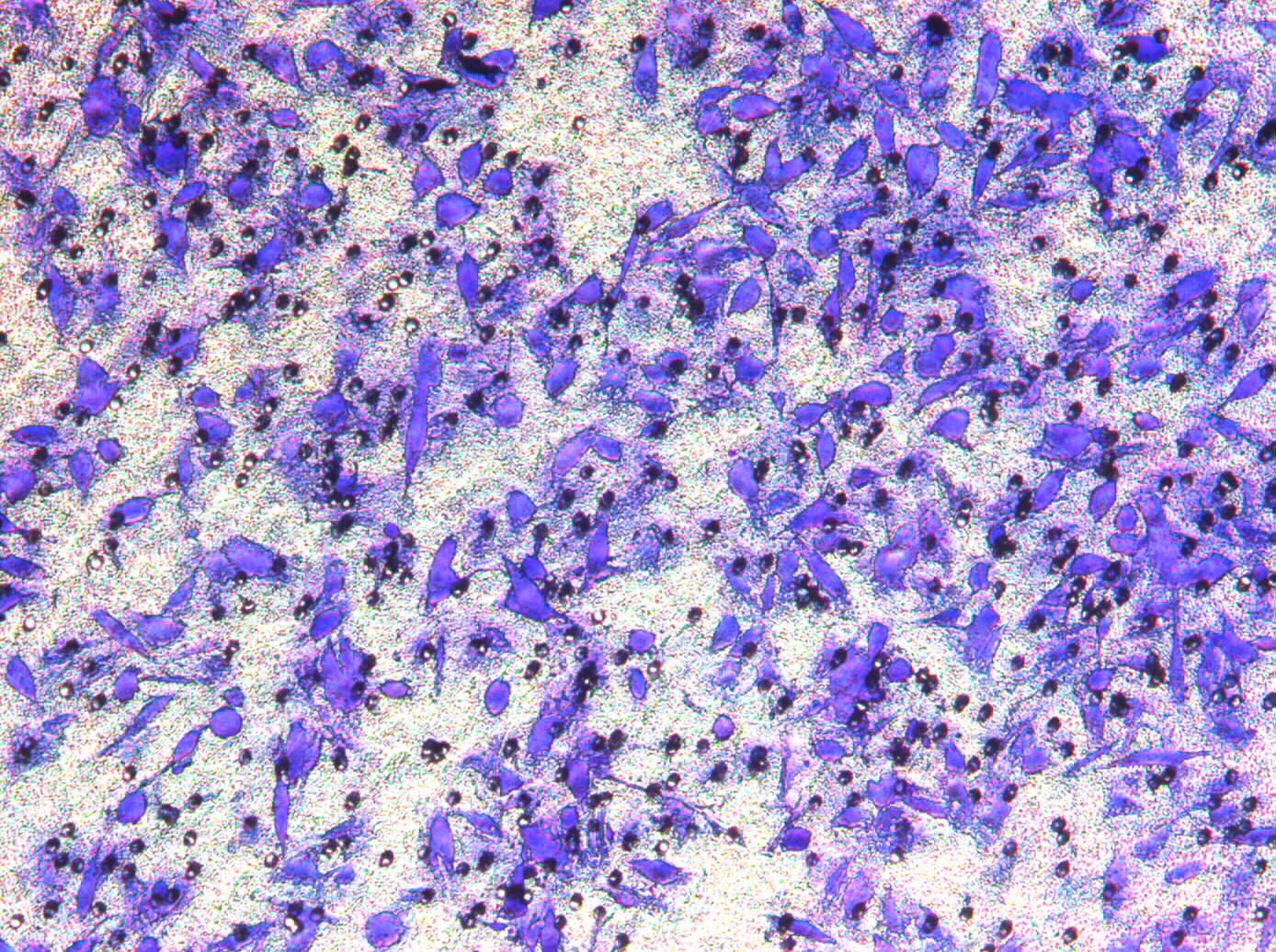

Supplement: S1 File — (ZIP) [file pone.0303435.s001.zip › File 3 - images for Figure 2D transwell/P30-1.jpg]

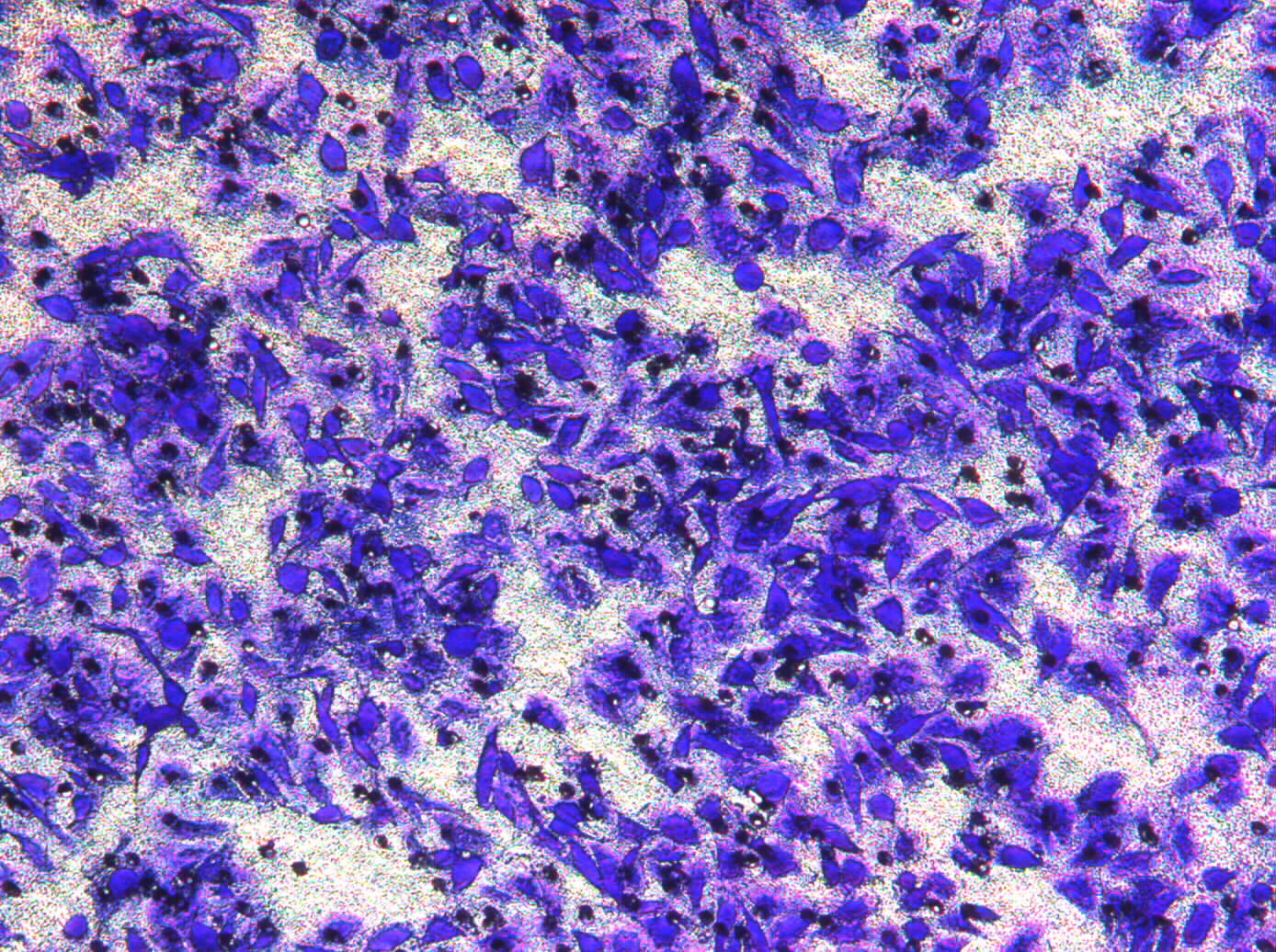

Supplement: S1 File — (ZIP) [file pone.0303435.s001.zip › File 3 - images for Figure 2D transwell/P30-2.jpg]

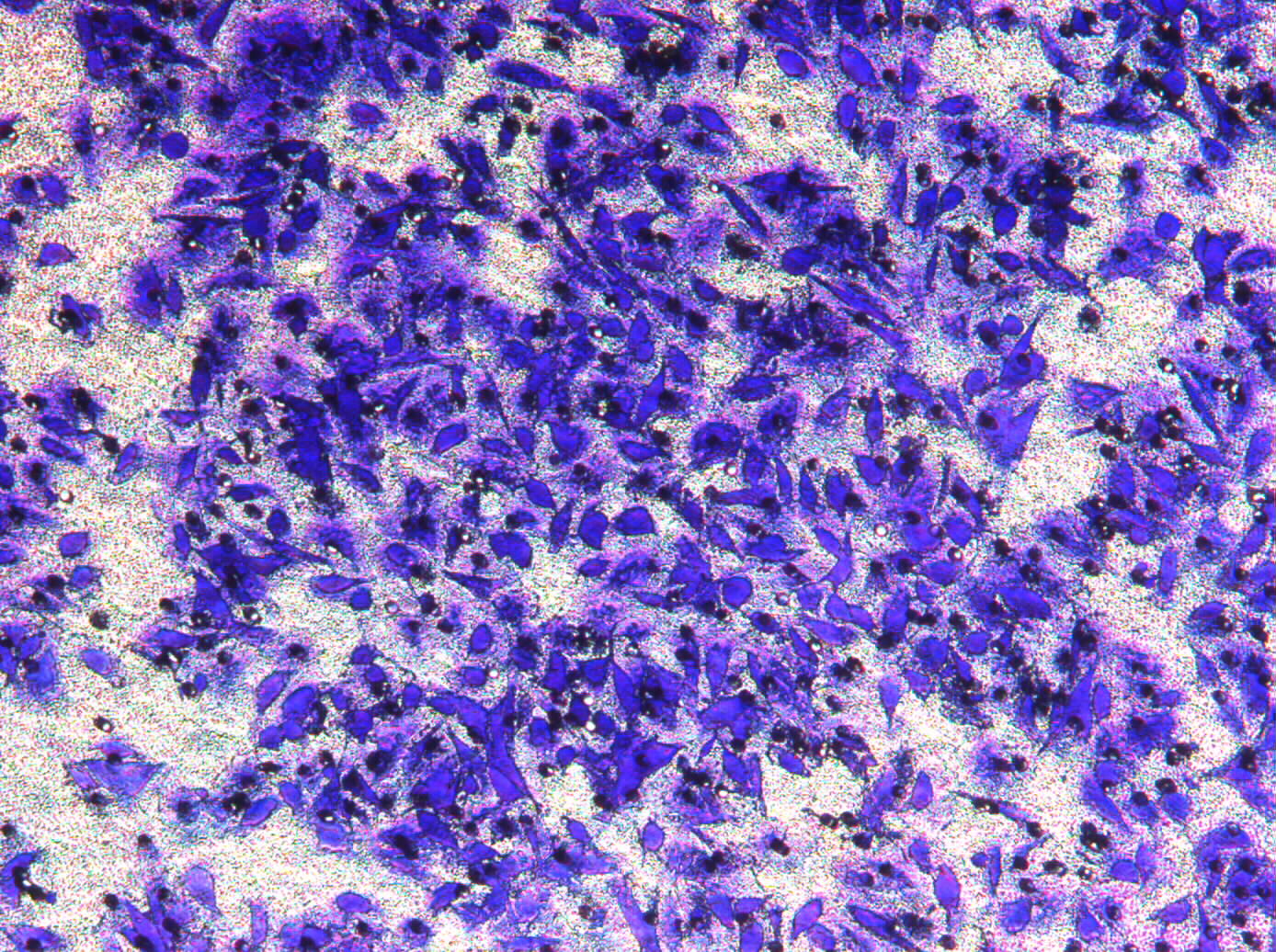

Supplement: S1 File — (ZIP) [file pone.0303435.s001.zip › File 3 - images for Figure 2D transwell/P30-3.jpg]

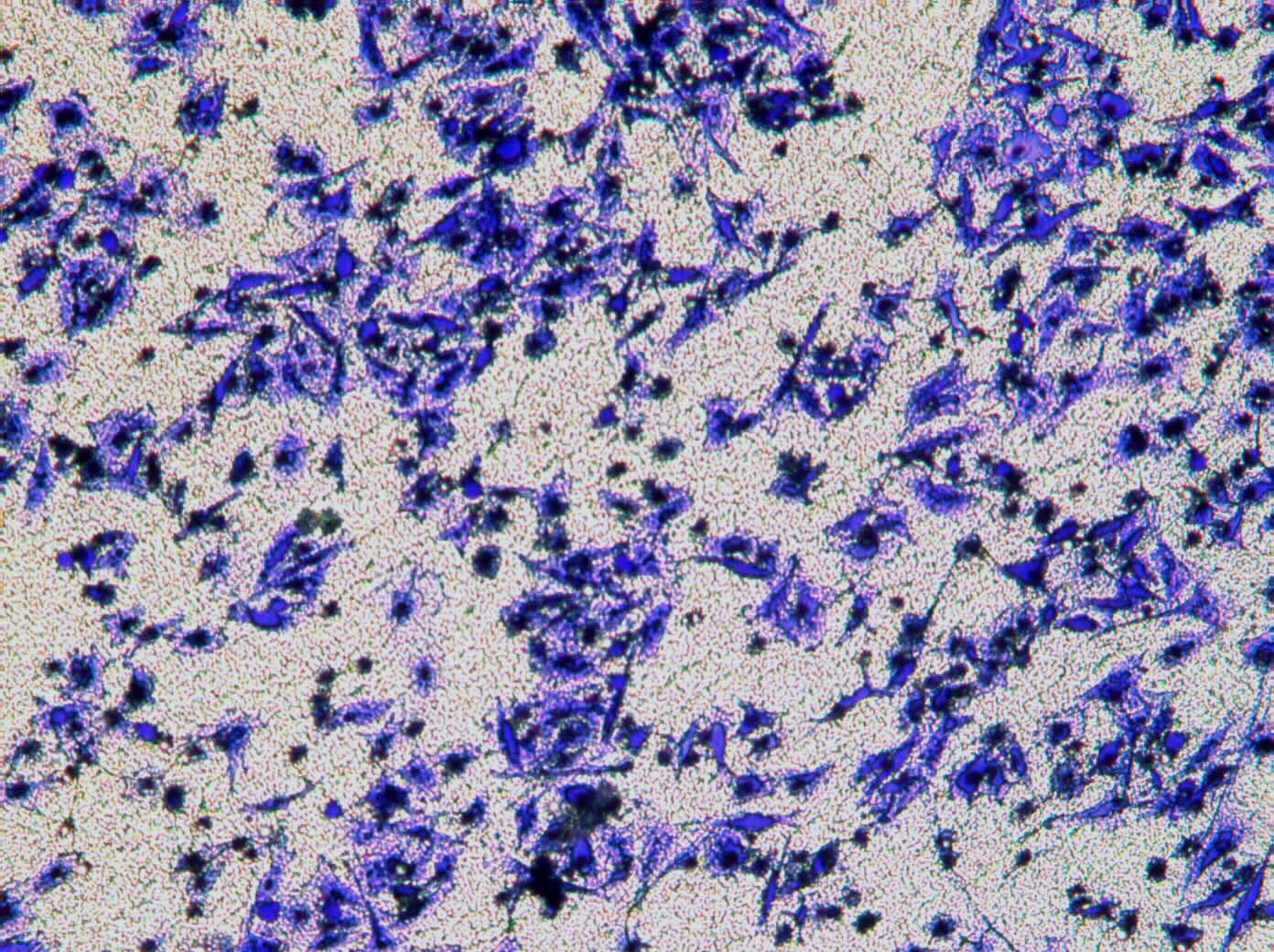

Supplement: S1 File — (ZIP) [file pone.0303435.s001.zip › File 3 - images for Figure 2D transwell/SC-1.jpg]

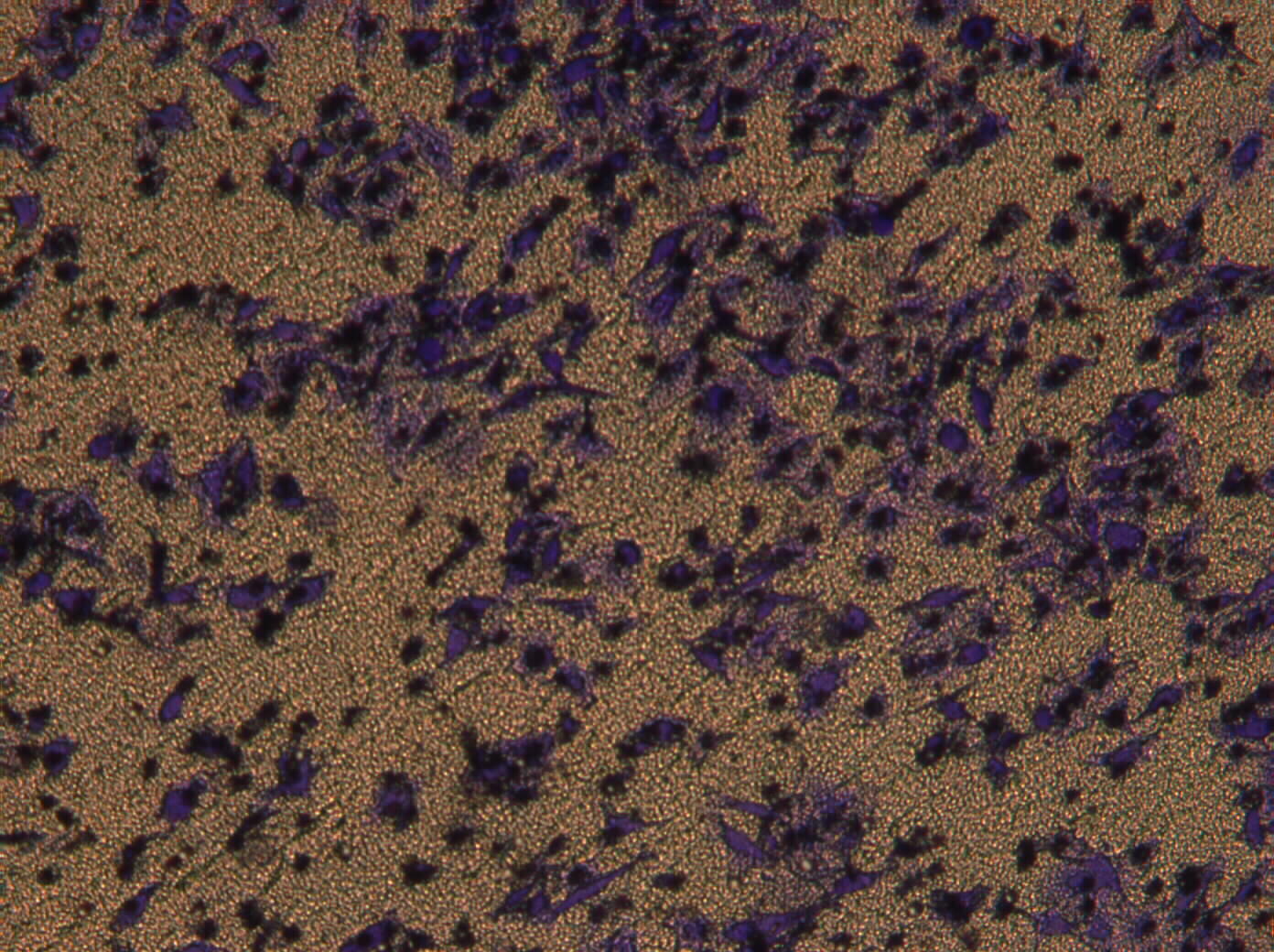

Supplement: S1 File — (ZIP) [file pone.0303435.s001.zip › File 3 - images for Figure 2D transwell/SC-2.jpg]

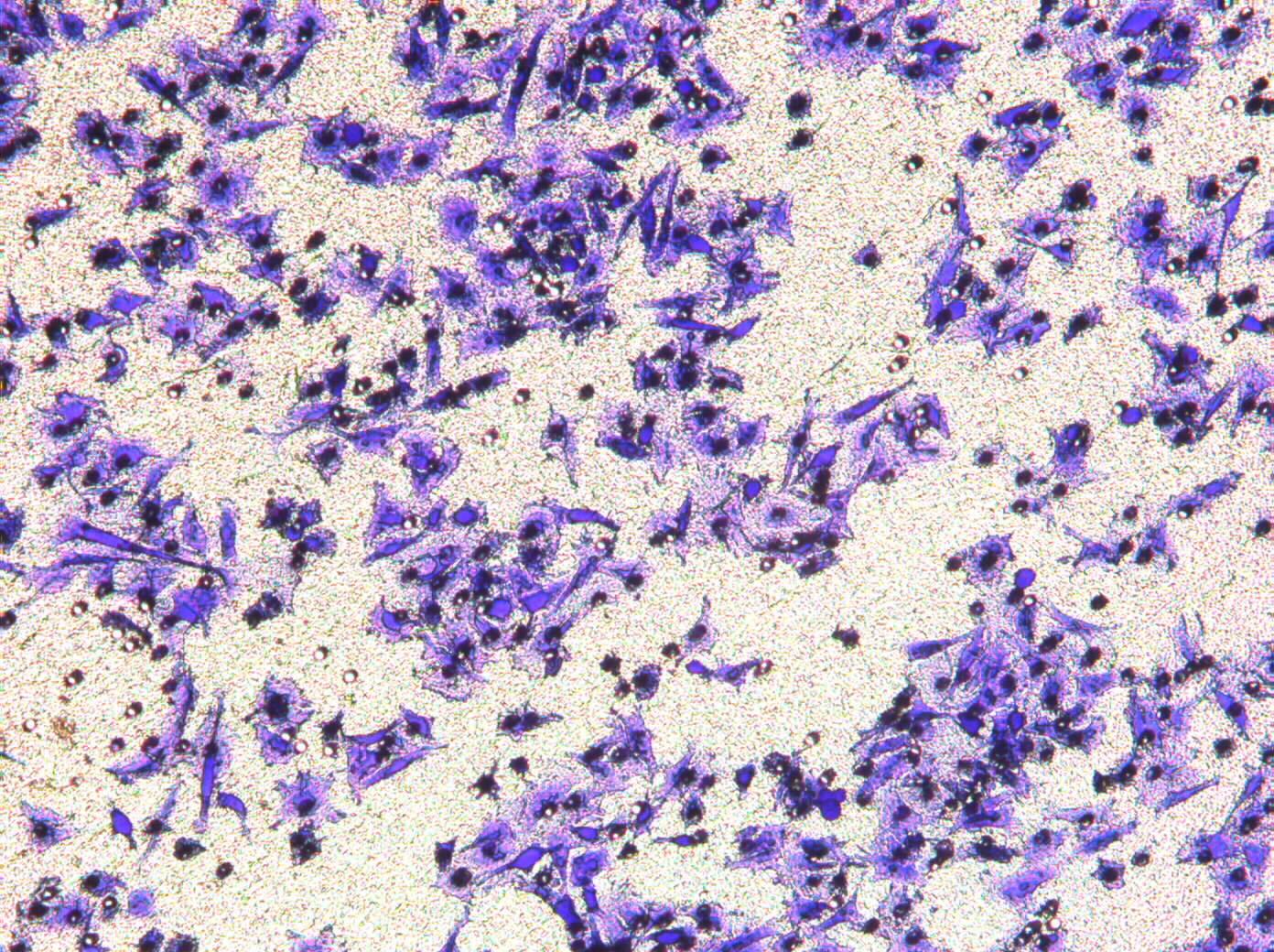

Supplement: S1 File — (ZIP) [file pone.0303435.s001.zip › File 3 - images for Figure 2D transwell/WT-1.jpg]

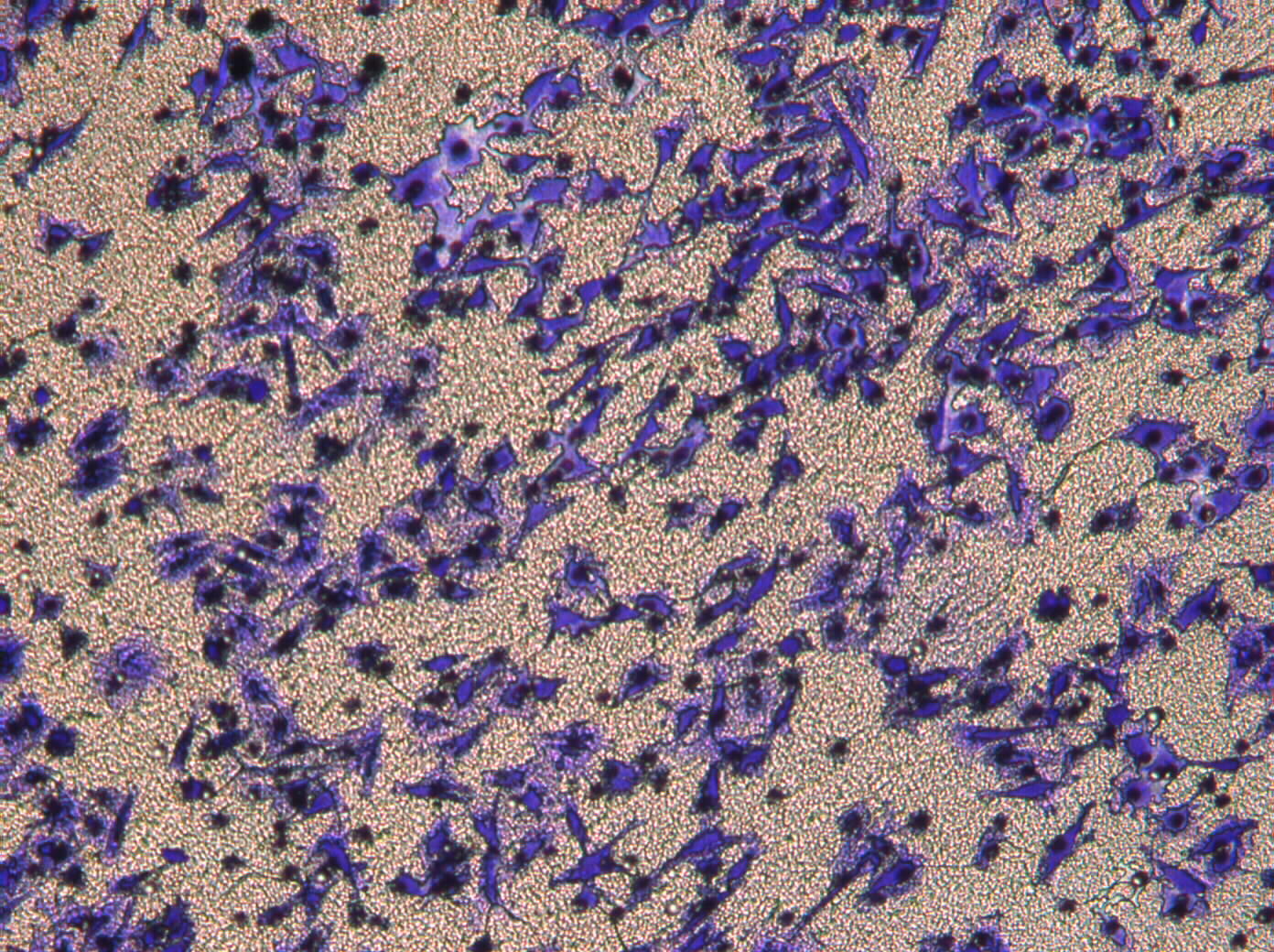

Supplement: S1 File — (ZIP) [file pone.0303435.s001.zip › File 3 - images for Figure 2D transwell/WT-2.jpg]

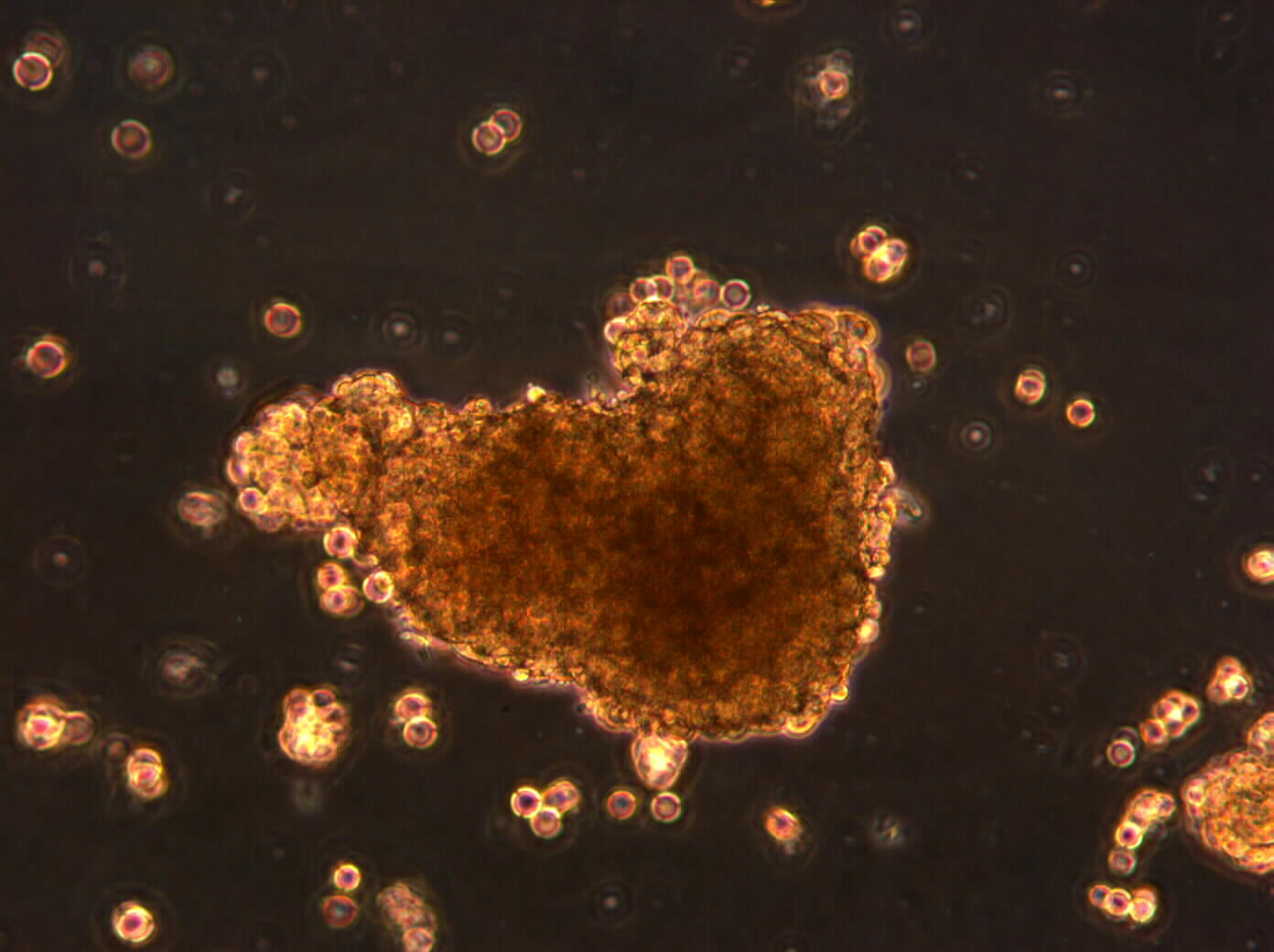

Supplement: S1 File — (ZIP) [file pone.0303435.s001.zip › File 4--Images for Figure 2F spheroid/G19.jpg]

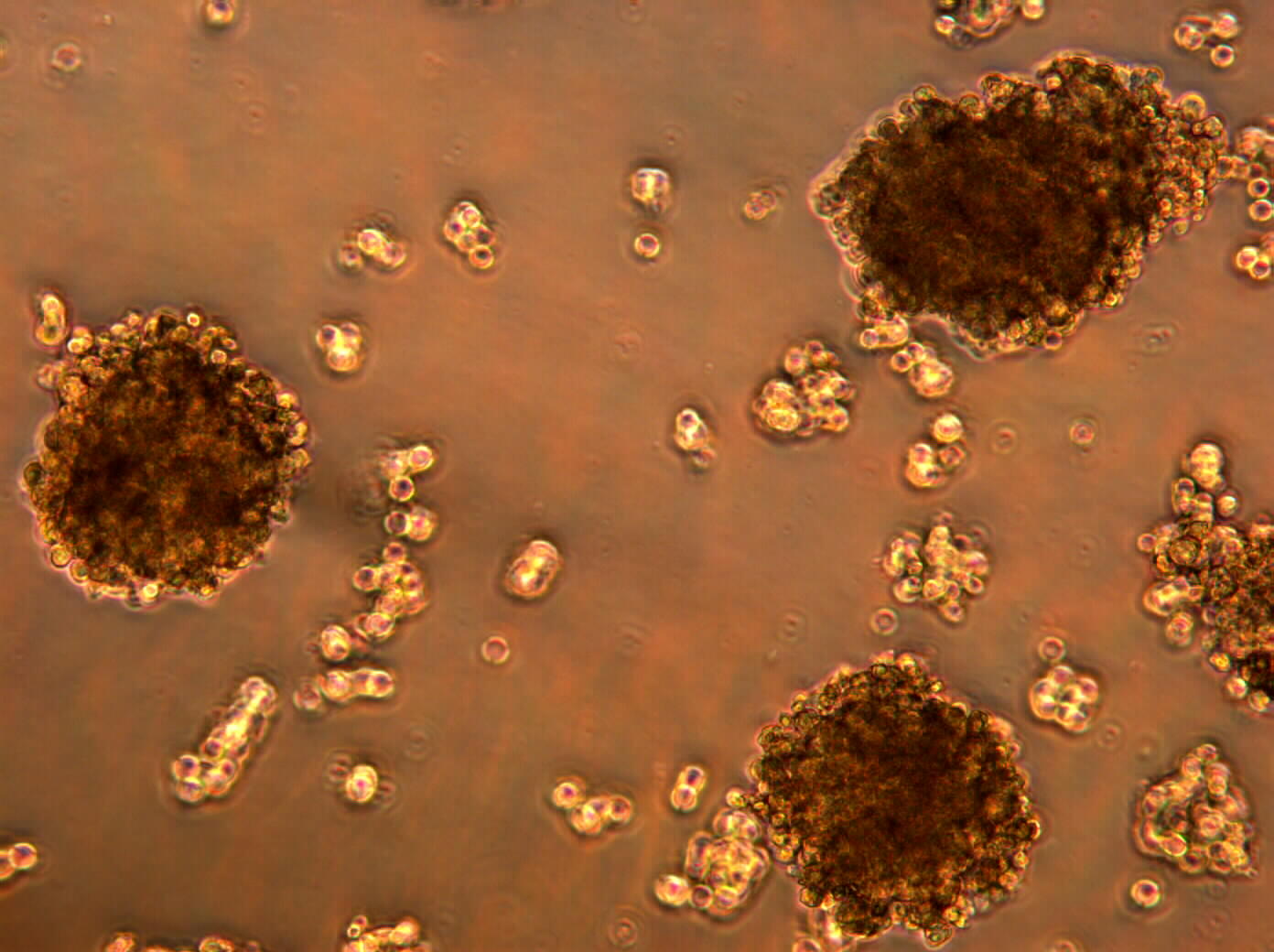

Supplement: S1 File — (ZIP) [file pone.0303435.s001.zip › File 4--Images for Figure 2F spheroid/P30.jpg]

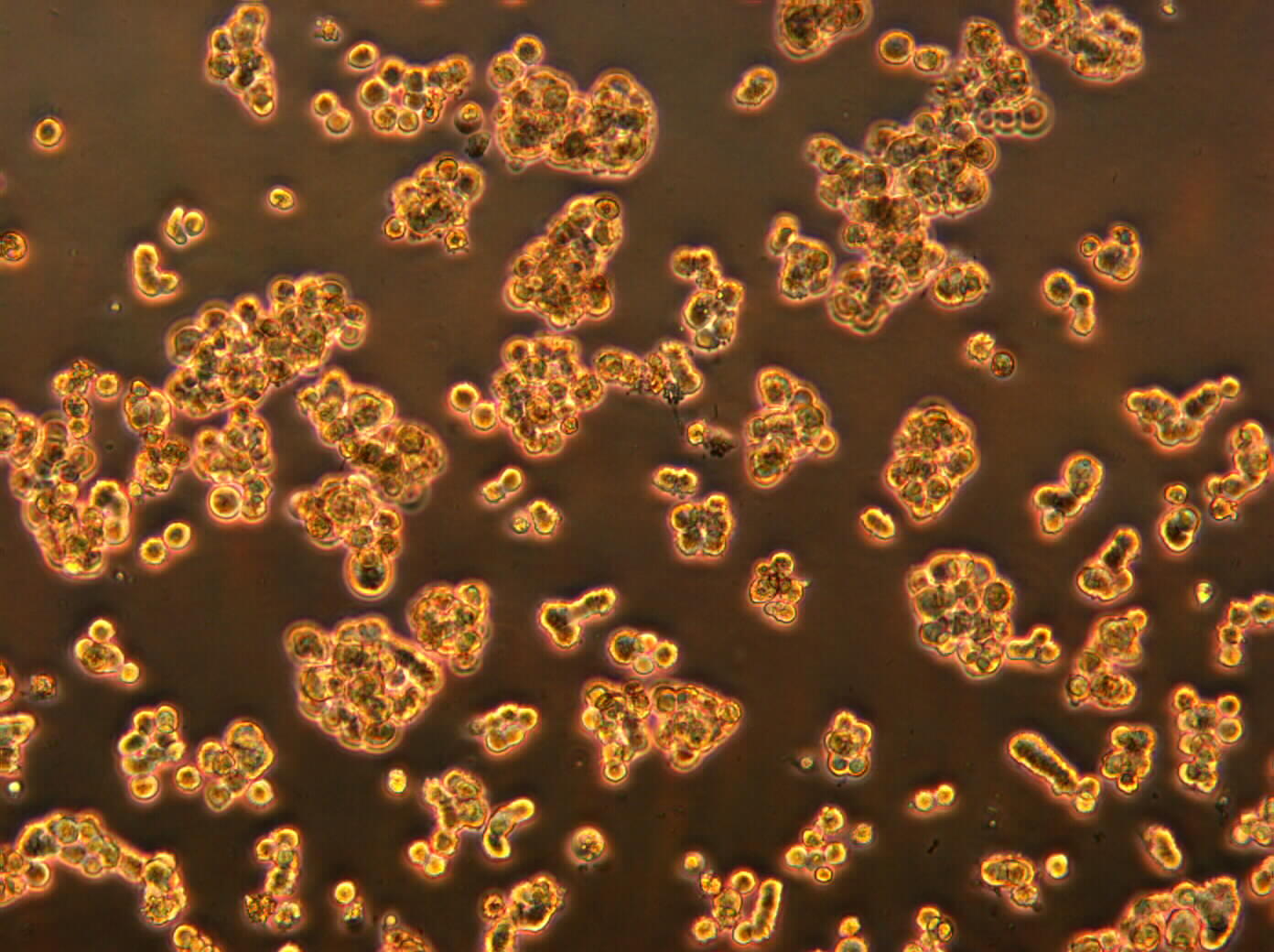

Supplement: S1 File — (ZIP) [file pone.0303435.s001.zip › File 4--Images for Figure 2F spheroid/SC.jpg]

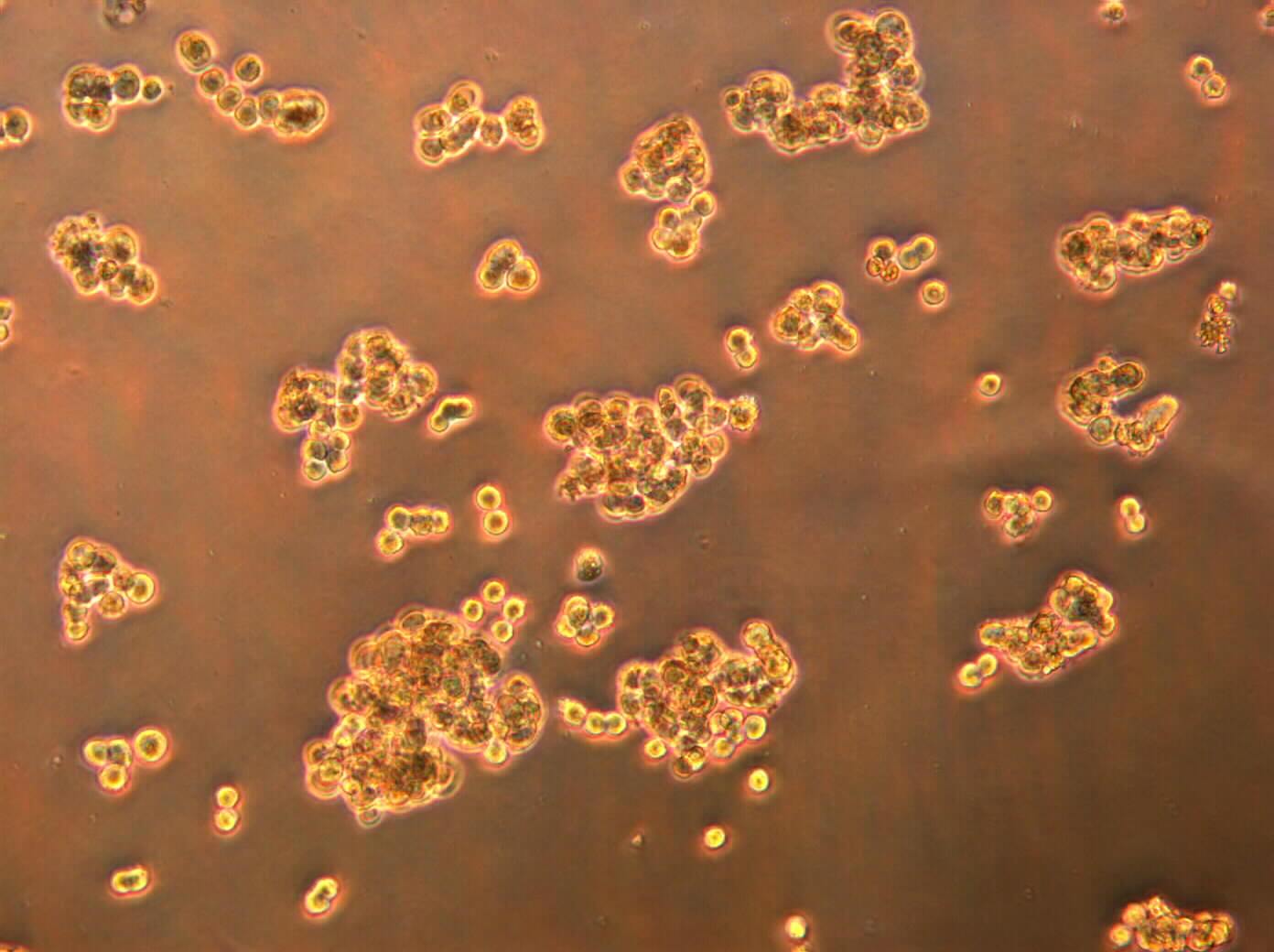

Supplement: S1 File — (ZIP) [file pone.0303435.s001.zip › File 4--Images for Figure 2F spheroid/WT.jpg]
